# Supplementary material for: A [3+3] Aldol-SNAr-Dehydration Approach to 2-Naphthol and 7-Hydroxyquinoline Derivatives
Source: Molecules. 2024 Jul 20;29(14):3406. doi: 10.3390/molecules29143406 (PMC11280476; doi:10.3390/molecules29143406)
Supplement: Supplementary file 1 [file molecules-29-03406-s001.zip › molecules-3108500-supplementary.pdf]

A [3+3] Aldol-SNAr-Dehydration Approach to 2-Naphthol and 7-Hydroxyquinoline Derivatives

Kwabena Fobi, Ebenezer Ametsetor and Richard A. Bunce *

Department of Chemistry, Oklahoma State University, Stillwater, OK 74078-3071, USA; kfobi@okstate.edu (K.F.); eametse@okstate.edu (E.A.)

* Correspondence: rab@okstate.edu; Tel.: +1-405-744-5952

Supplemental Information

Compound **Page**

NMR data for methyl 3-oxo-4-phenylbutanoate (**8**)……………………………………………3
NMR data for methyl 4-(2-fluorophenyl)-3-oxobutanoate (**9**)………………………………4
NMR data for methyl 4-(4-chlorophenyl)-3-oxobutanoate (**10**)………………………………5
NMR data for methyl 4-(4-methylphenyl)-3-oxobutanoate (**11**)……………………………… 6
NMR data for methyl 4-(4-methoxyphenyl)-3-oxobutanoate (**12**)…………………………… …7
NMR data for methyl 3-oxo-4-(3-(trifluoromethyl)phenyl)butanoate (**13**)…………………… 8
NMR data for methyl 4-(2,5-dimethylphenyl)-3-oxobutanoate (**14**)………………………… 9
NMR data for 1,3-dibenzoylpropan-2-one (**15**)………………………………………………10
NMR data for 1-phenyl-3-(phenylsulfonyl)propan-2-one (**16**)………………………………11
NMR data for 1,3-bis(phenylsulfonyl)propan-2-one (**17**)……………………………………...12
NMR data for dimethyl 2-hydroxy-6-nitronaphthalene-1,3-dicarboxylate (**18**)………………13
NMR data for diethyl 2-hydroxy-6-nitronaphthalene-1,3-dicarboxylate (**19**)…………………14
NMR data for methyl 3-hydroxy-7-nitro-4-phenyl-2-naphthoate (**20**)…………………………15
NMR data for methyl 4-(2-fluorophenyl)-3-hydroxy-7-nitro-2-naphthoate (**21**)………………16
NMR data for methyl 4-(4-chlorophenyl)-3-hydroxy-7-nitro-2-naphthoate (**22**)………………17
NMR data for methyl 3-hydroxy-4-(4-methylphenyl)-7-nitro-2-naphthoate (**23**)………………18
NMR data for methyl 3-hydroxy-4-(4-methoxyphenyl)-7-nitro-2-naphthoate (**24**)……………..19
NMR data for methyl 3-hydroxy-7-nitro-4-(3-(trifluoromethyl)phenyl)-2-naphthoate (**25**)…….20
NMR data of methyl 4-(2,5-dimethylphenyl)-3-hydroxy-7-nitro-2-naphthoate (**26**)……………21
NMR data of 1,3-dibenzoyl-3-hydroxy-6-nitro-2-naphthoate (**27**)……………………………22
NMR data for 6-nitro-1-phenyl-3-(phenylsulfonyl)naphthalen-2-ol (**28**)……………………… 23
NMR data for 5-nitro-2-(phenylsulfonyl)benzaldehyde (**29**)………………………………… 24
NMR data of dimethyl 6-cyano-2-hydroxynaphthalene-1,3-dicarboxylate (**30**)…………………25
NMR data for methyl 7-cyano-3-hydroxy-4-phenyl-2-naphthoate (**31**)…………………………26
NMR data for methyl 7-cyano-4-(2-fluorophenyl)-3-hydroxy-2-naphthoate (**32**)………………27
NMR data for methyl 4-(4-chlorophenyl)-7-cyano-3-hydroxy-2-naphthoate (**33**)………………28
NMR data for methyl 7-cyano-3-hydroxy-4-(4-methoxyphenyl)-2-naphthoate (**34**)……………29

NMR data for methyl 7-cyano-4-(2,5-dimethylphenyl)-3-hydroxy-2-naphthoate (**35**)…..………30
NMR data for 5,7-dibenzoyl-6-hydroxy-2-naphthonitrile (**36**)…….………………………….…31
NMR data for ethyl (*E*)-3-(2-fluoro-5-(trifluoromethyl)phenyl)acrylate (**37**)………………..….32
NMR data for methyl 4-(4-methylphenyl)-3-hydroxy-7-(trifluoromethyl)-2-naphthoate (**38**)….33
NMR data for methyl 3-hydroxy-4-(4-methoxyphenyl)-7-(trifluoromethyl)-2-naphthoate (**39**)..34
NMR data for ethyl (*E*)-3-(2-fluoropyridin-3-yl)acrylate (**40**)……………………………….…35
NMR data for methyl 7-hydroxy-8-phenylquinoline-6-carboxylate (**41**)……………………..….36
NMR data for methyl 8-(4-chlorophenyl)-7-hydroxyquinoline-6-carboxylate (**42**)…………….…37
NMR data for methyl 8-(4-methylphenyl)-7-hydroxyquinoline-6-carboxylate (**43**)…….……....38
NMR data for methyl 7-hydroxy-8-(4-methoxyphenyl)quinoline-6-carboxylate (**44**)…………. 39

NMR data for methyl 3-oxo-4-phenylbutanoate (**8**)

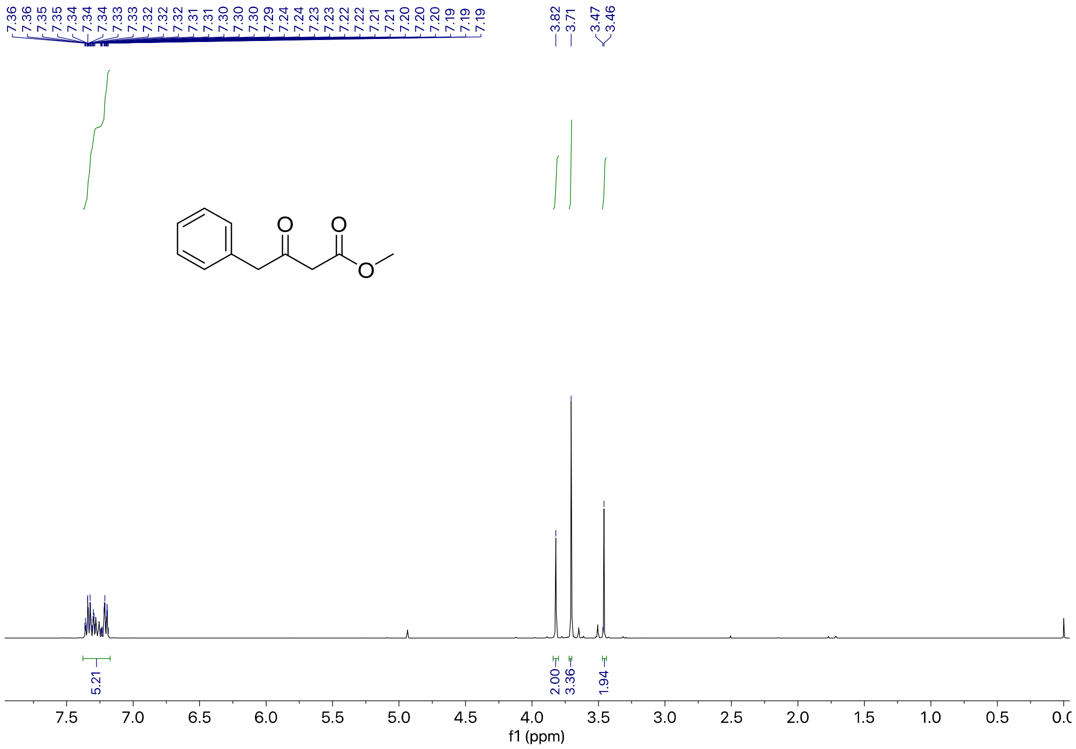

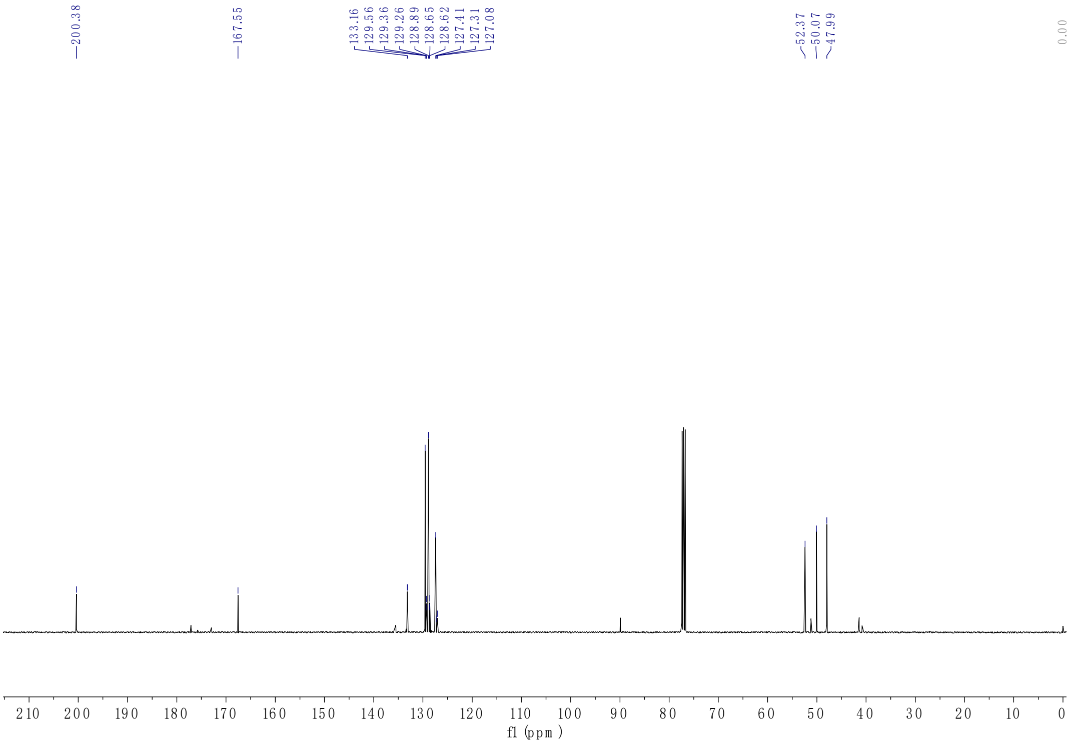

NMR data for methyl 4-(2-fluorophenyl)-3-oxobutanoate (**9**)

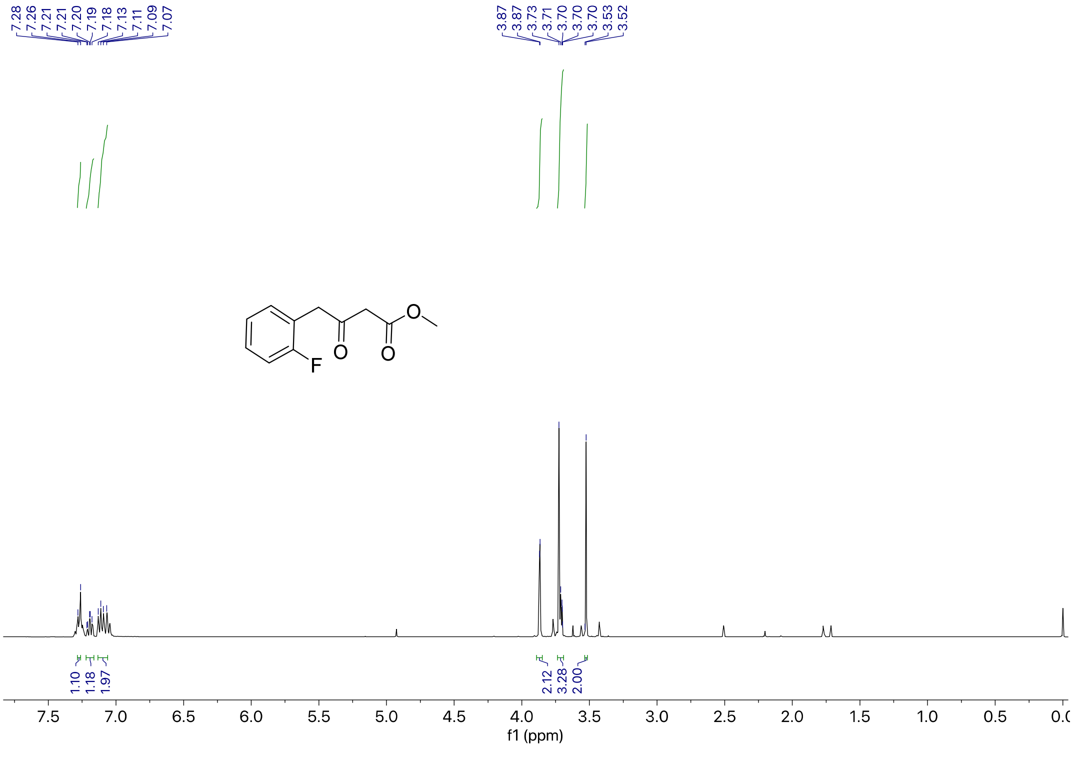

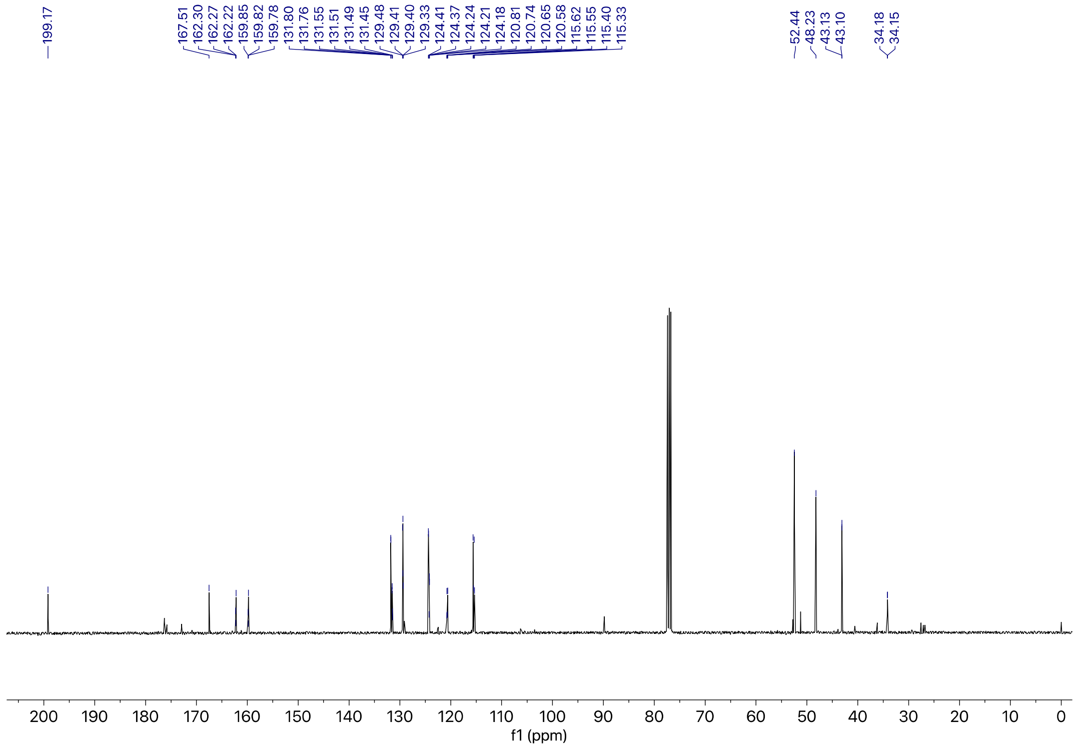

NMR data for methyl 4-(4-chlorophenyl)-3-oxobutanoate (**10**)

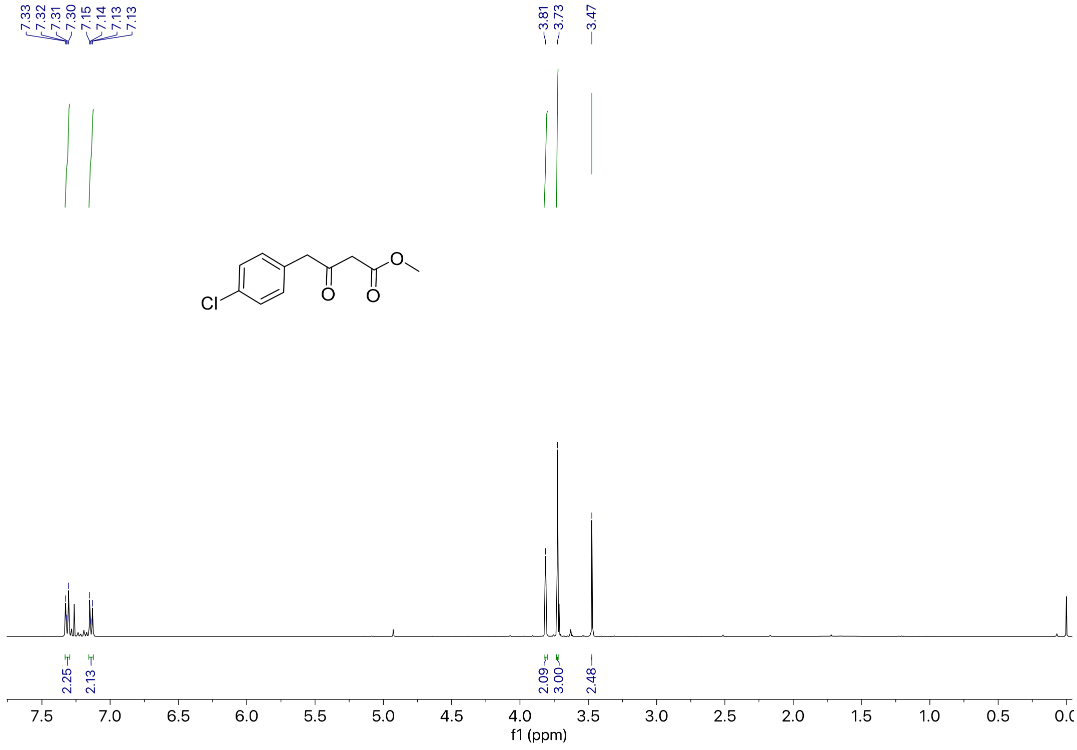

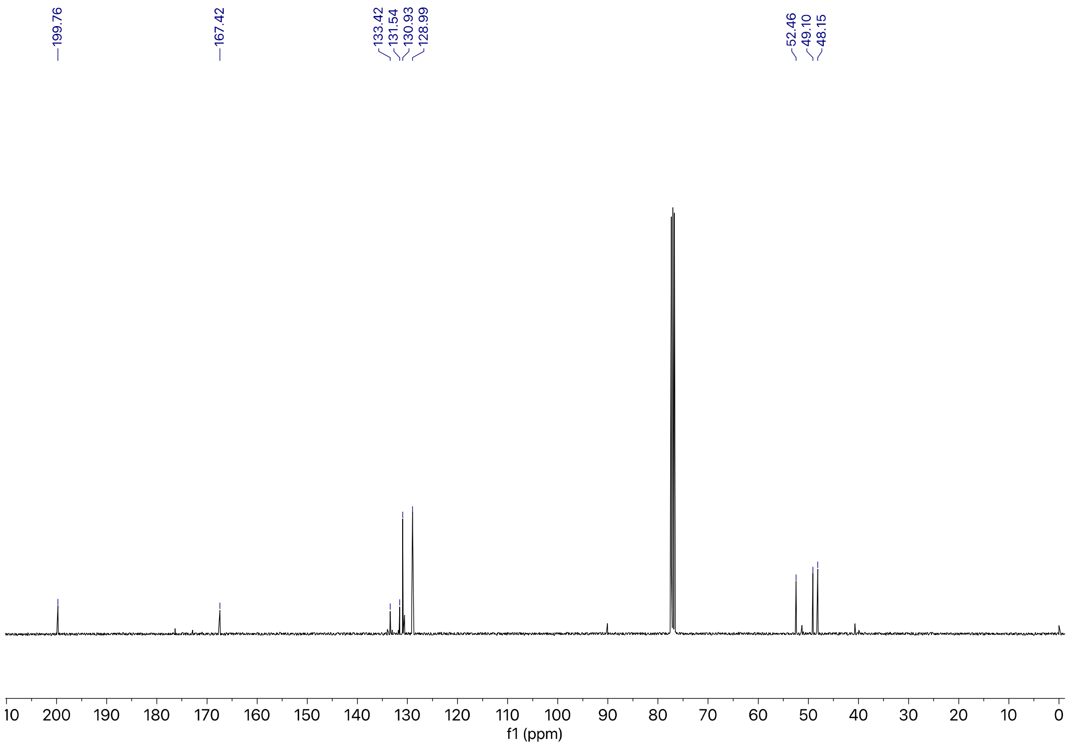

NMR data for methyl 4-(4-methylphenyl)-3-oxobutanoate (**11**)

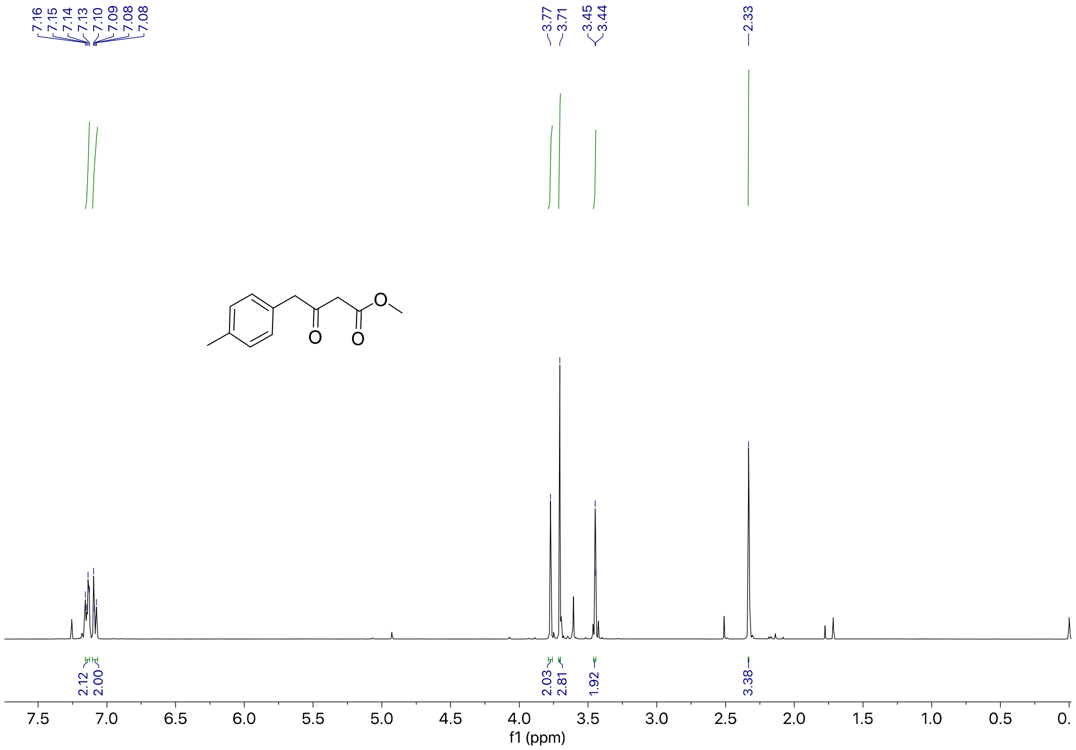

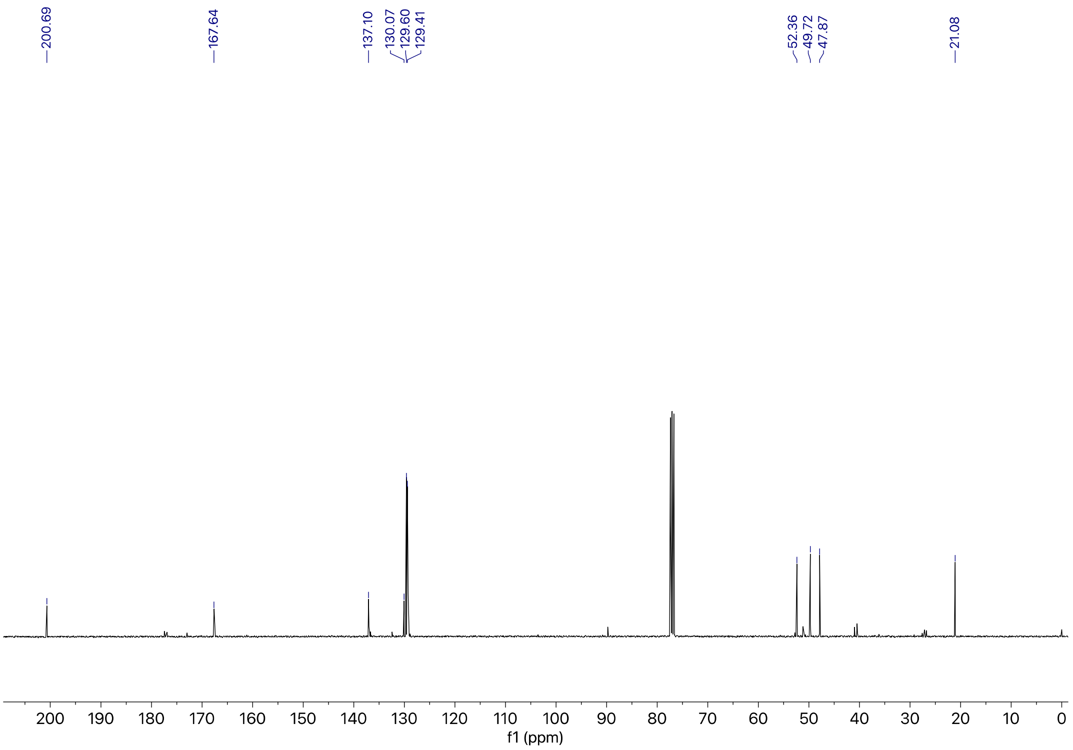

NMR data for methyl 4-(4-methoxyphenyl)-3-oxobutanoate (**12**)

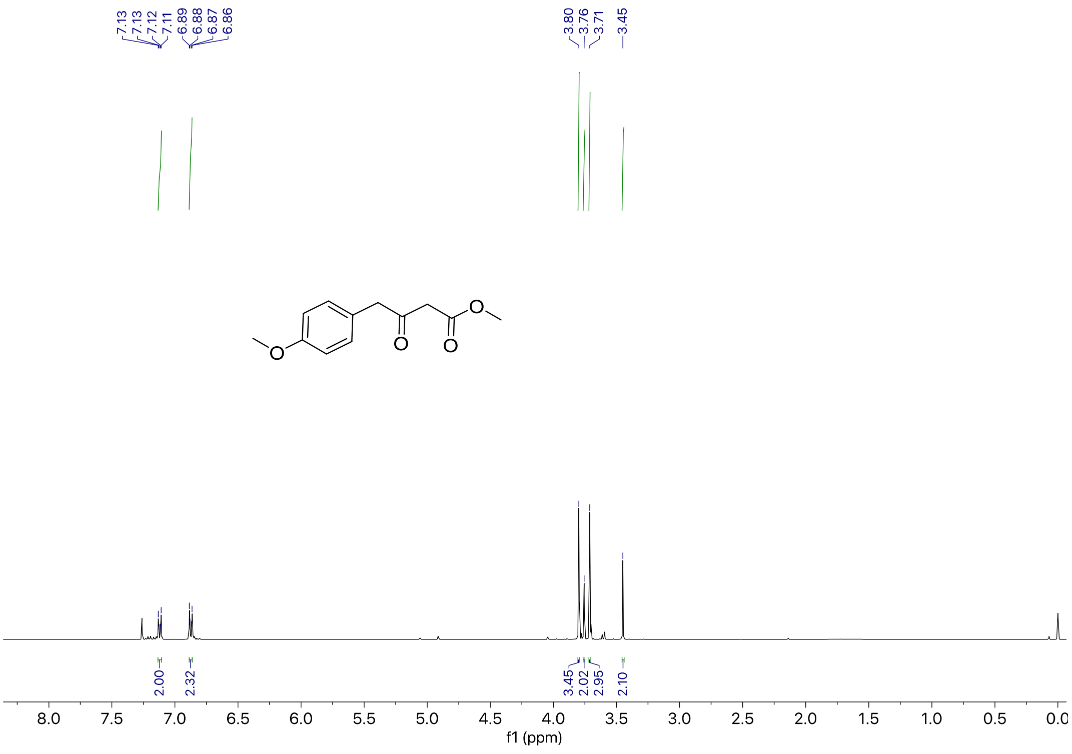

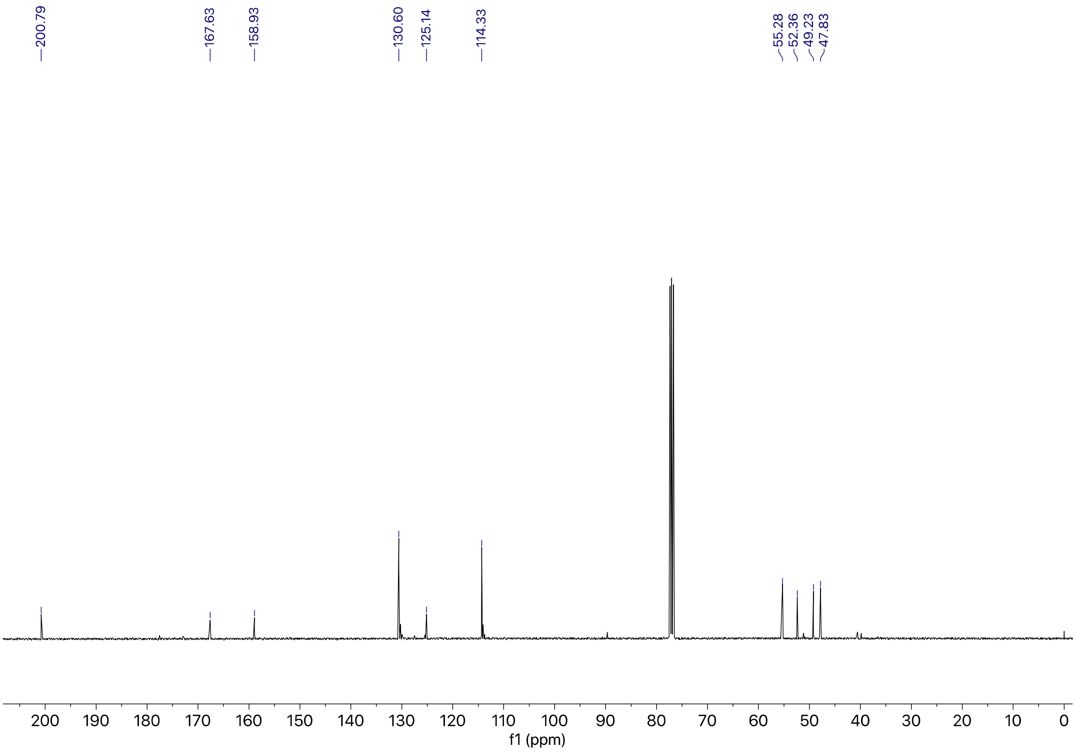

NMR data for methyl 3-oxo-4-(3-(trifluoromethyl)phenyl)butanoate (**13**)

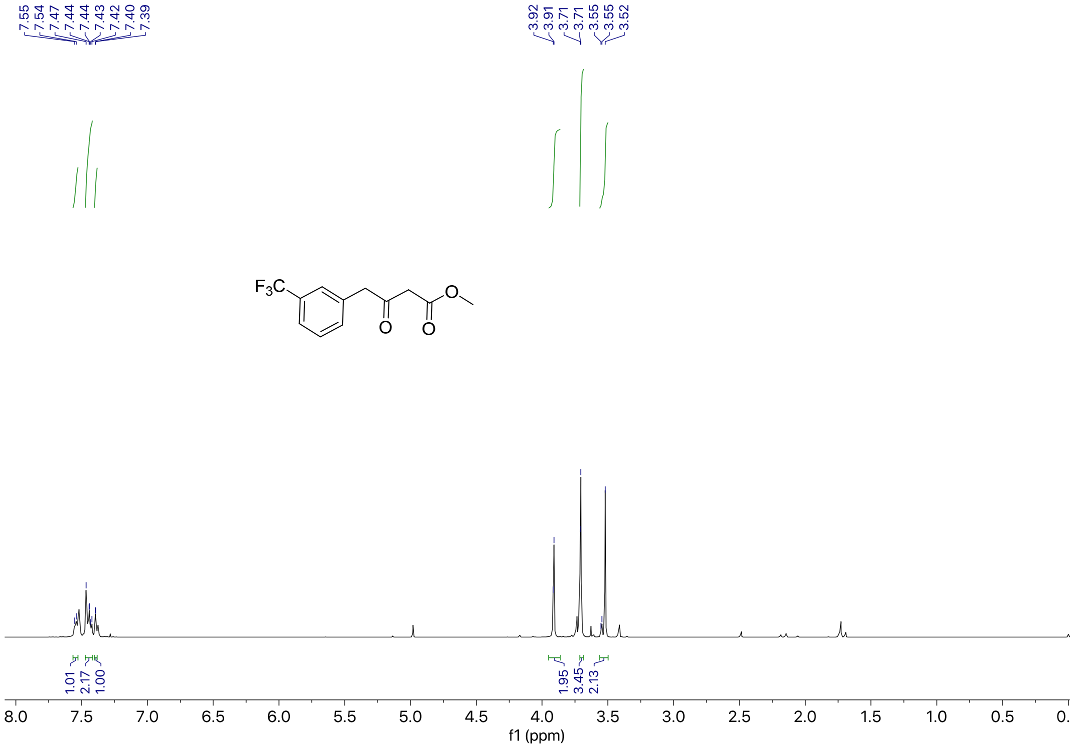

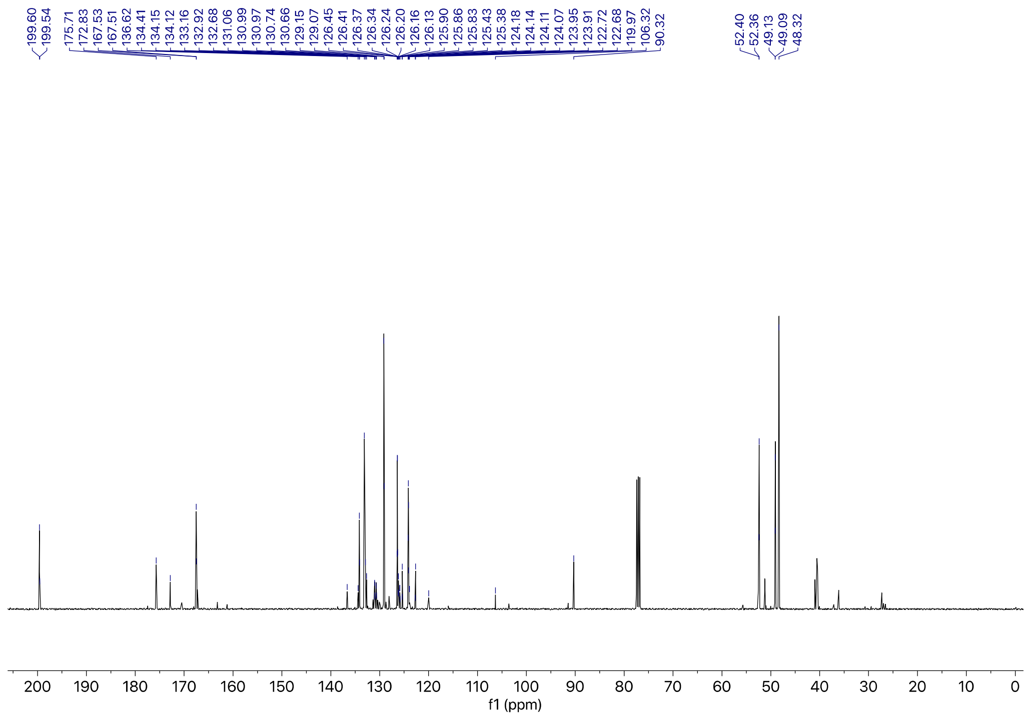

NMR data for methyl 4-(2,5-dimethylphenyl)-3-oxobutanoate (**14**)

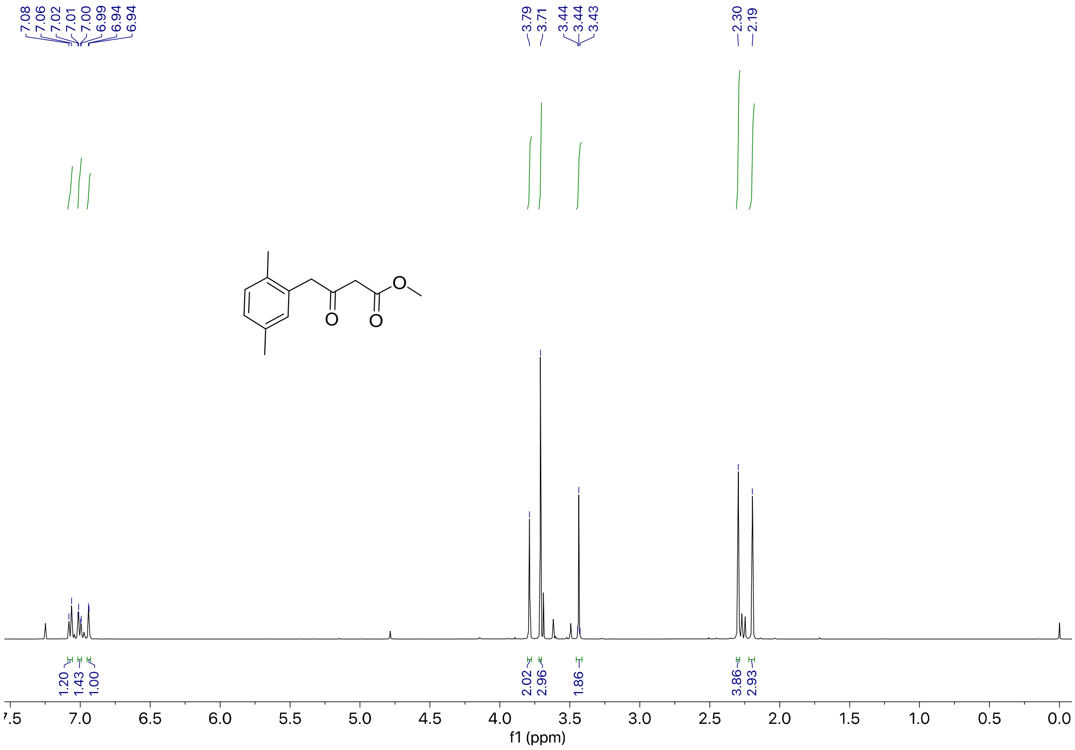

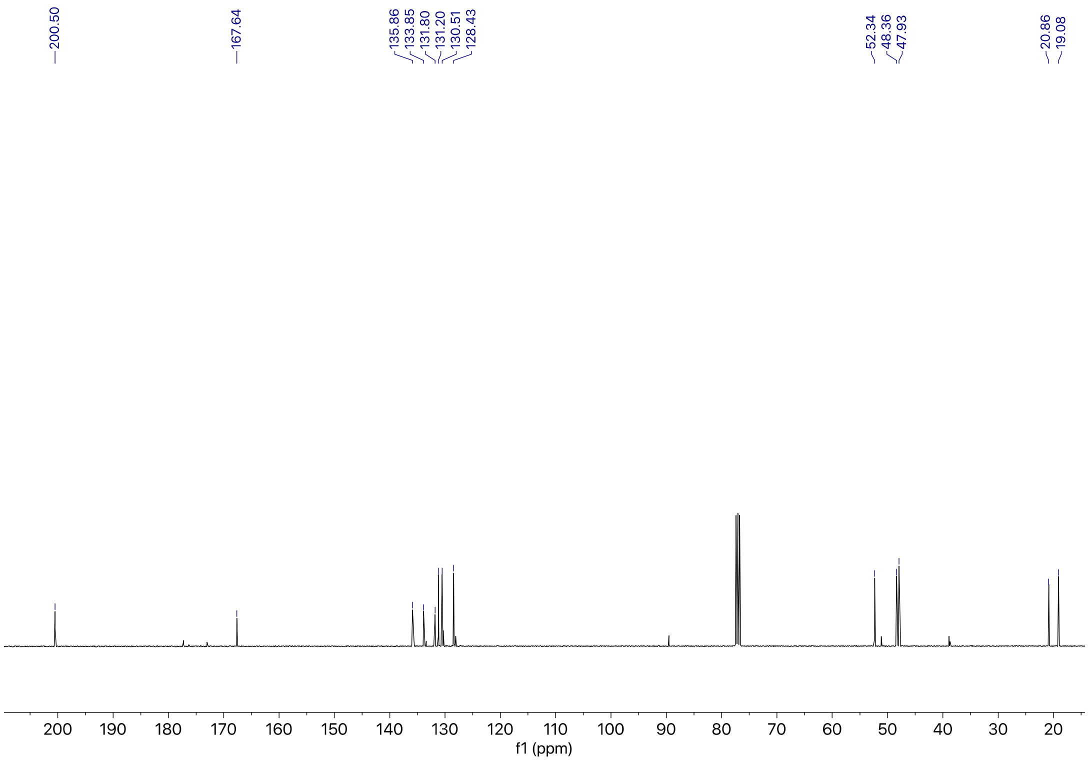

NMR data for 1,3-dibenzoylpropan-2-one (mostly enol, **15**)

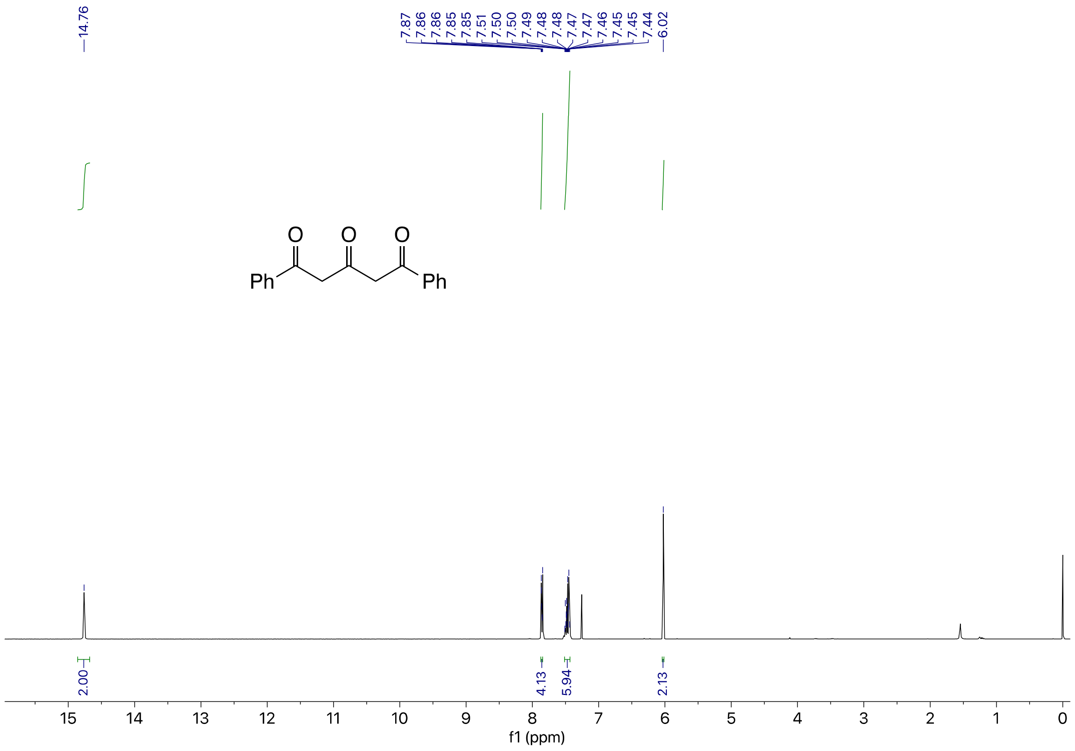

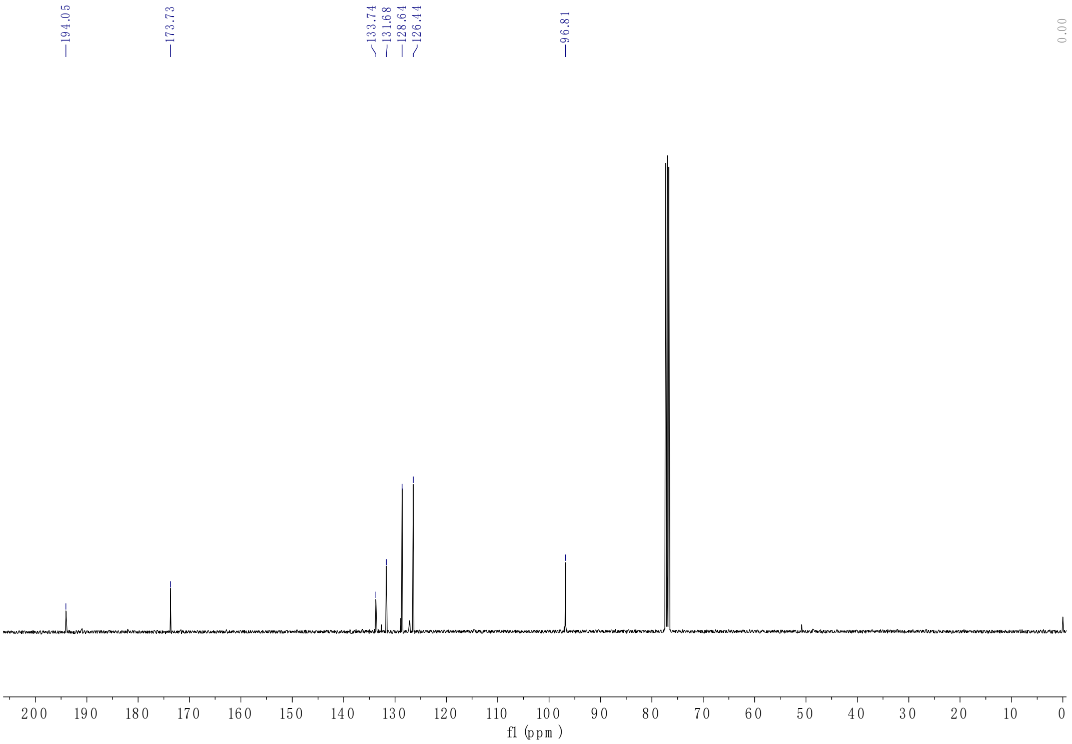

NMR data for 1-phenyl-3-(phenylsulfonyl)propan-2-one (**16**)

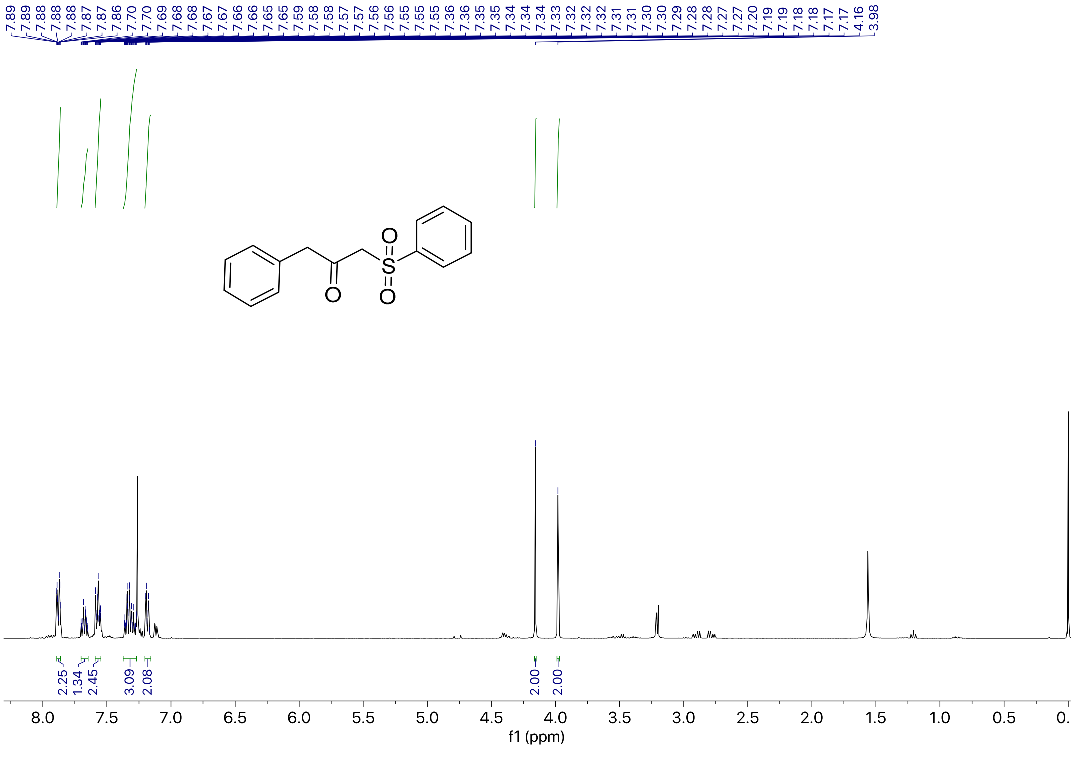

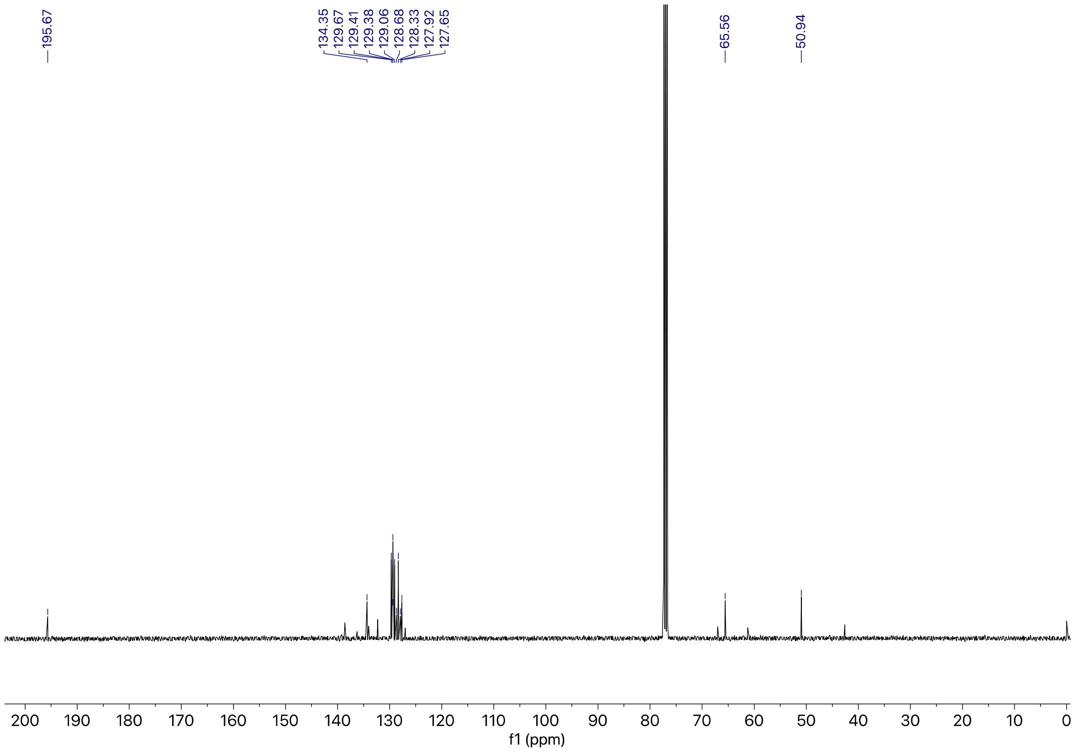

NMR data for 1,3-bis(phenylsulfonyl)propan-2-one (**17**)

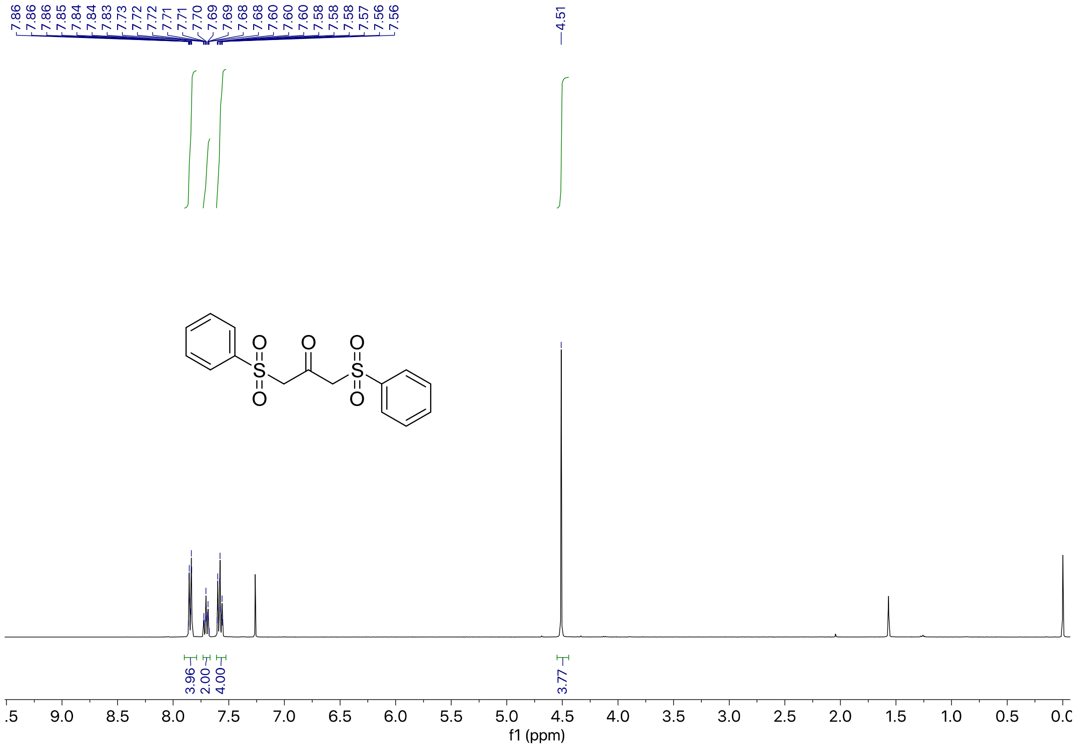

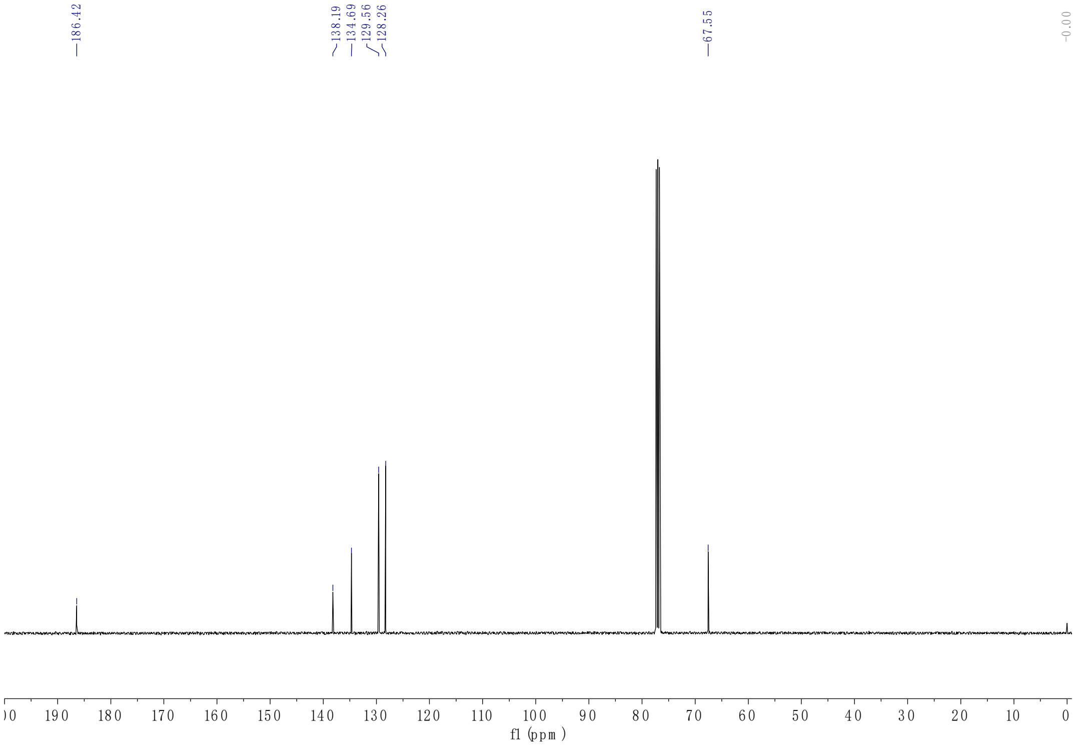

NMR data for dimethyl 2-hydroxy-6-nitronaphthalene-1,3-dicarboxylate (**18**)

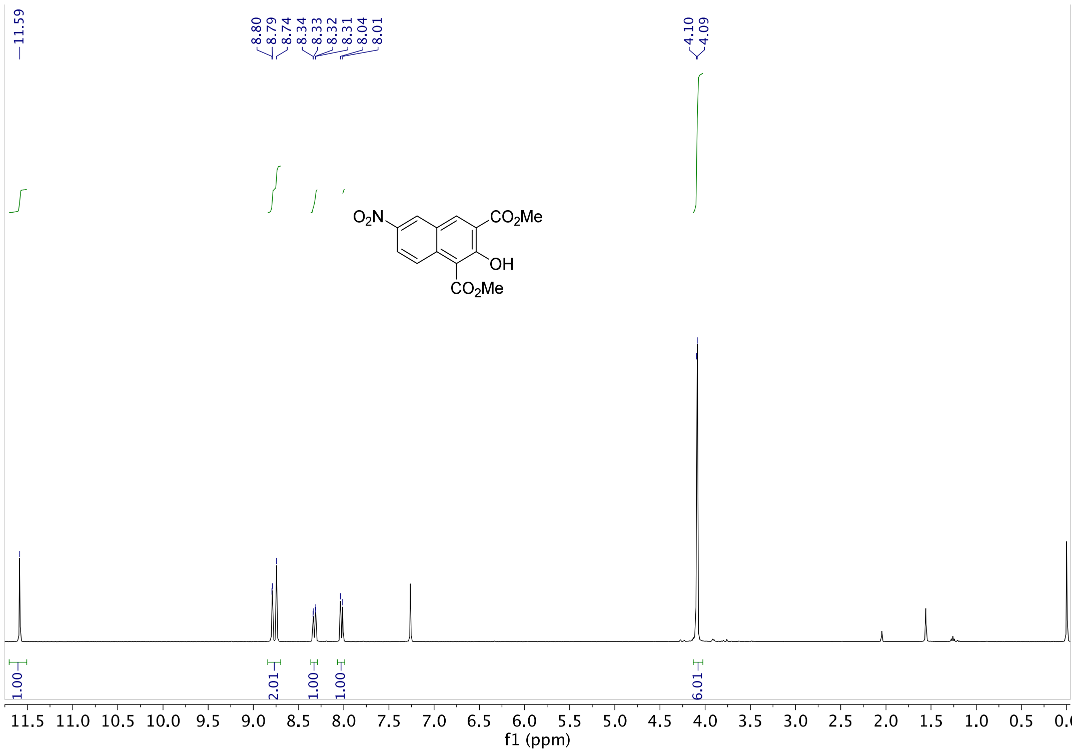

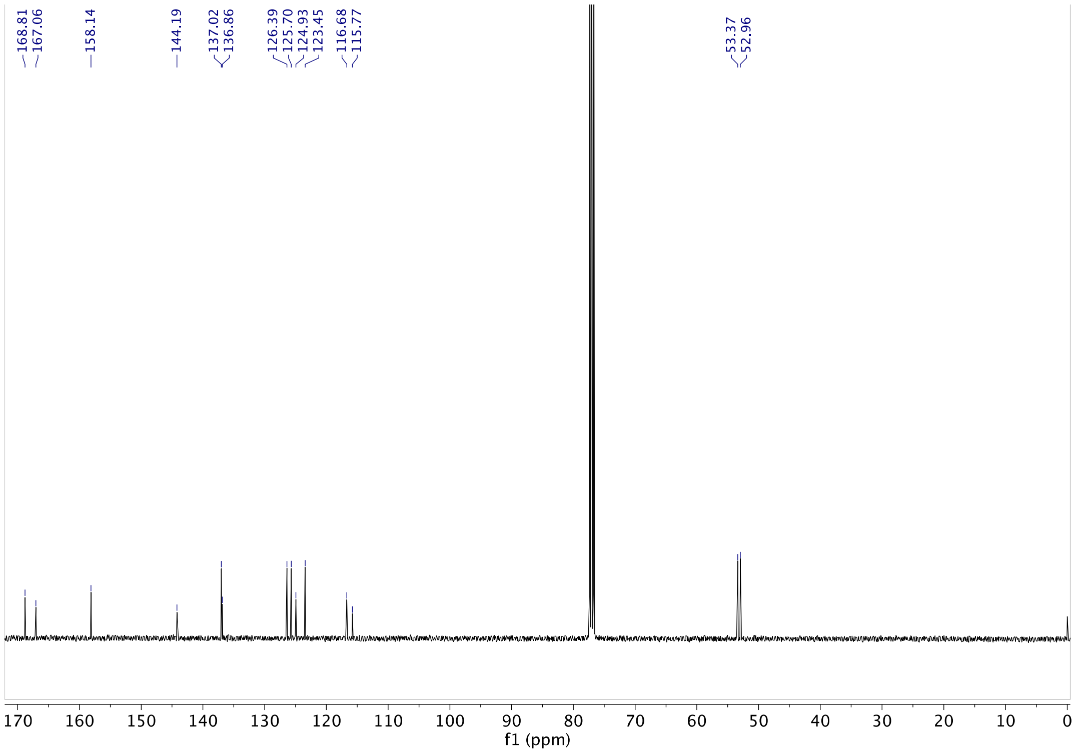

NMR data for diethyl 2-hydroxy-6-nitronaphthalene-1,3-dicarboxylate (**19**)

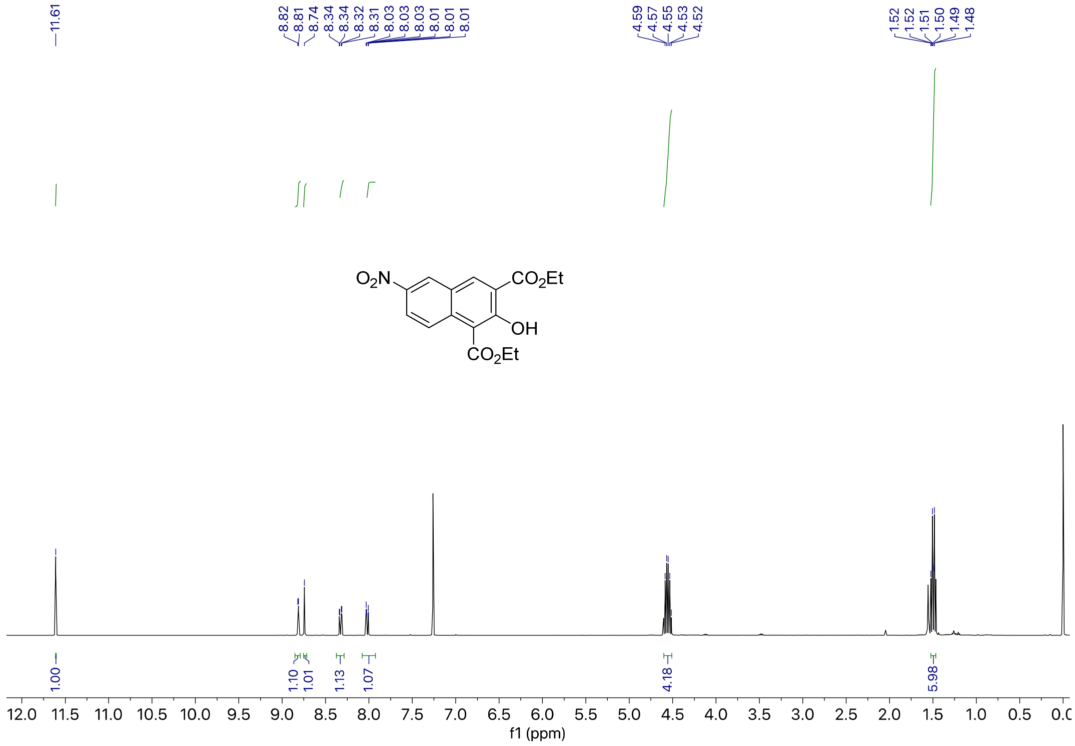

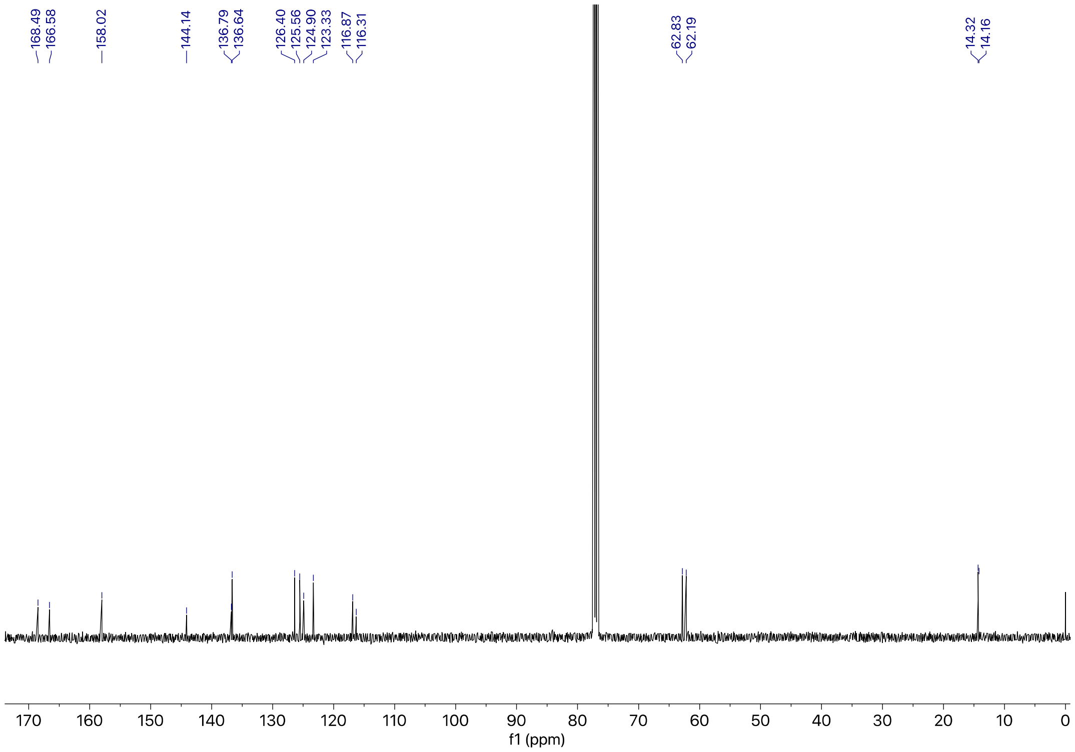

NMR data for methyl 3-hydroxy-7-nitro-4-phenyl-2-naphthoate (**20**)

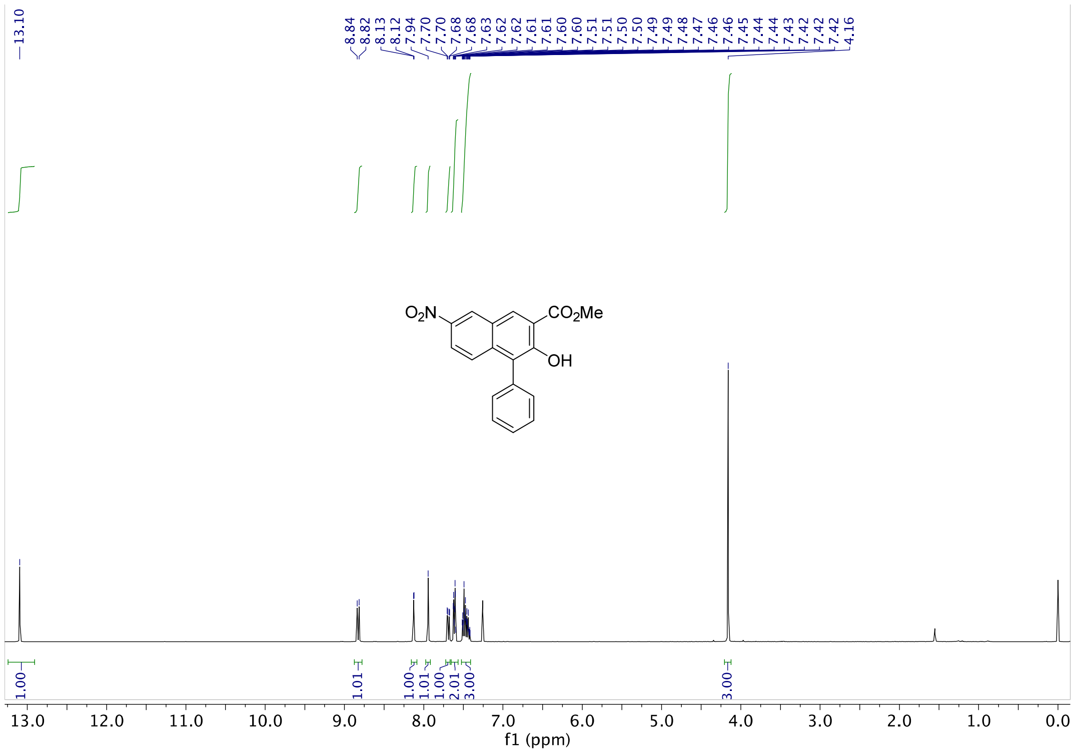

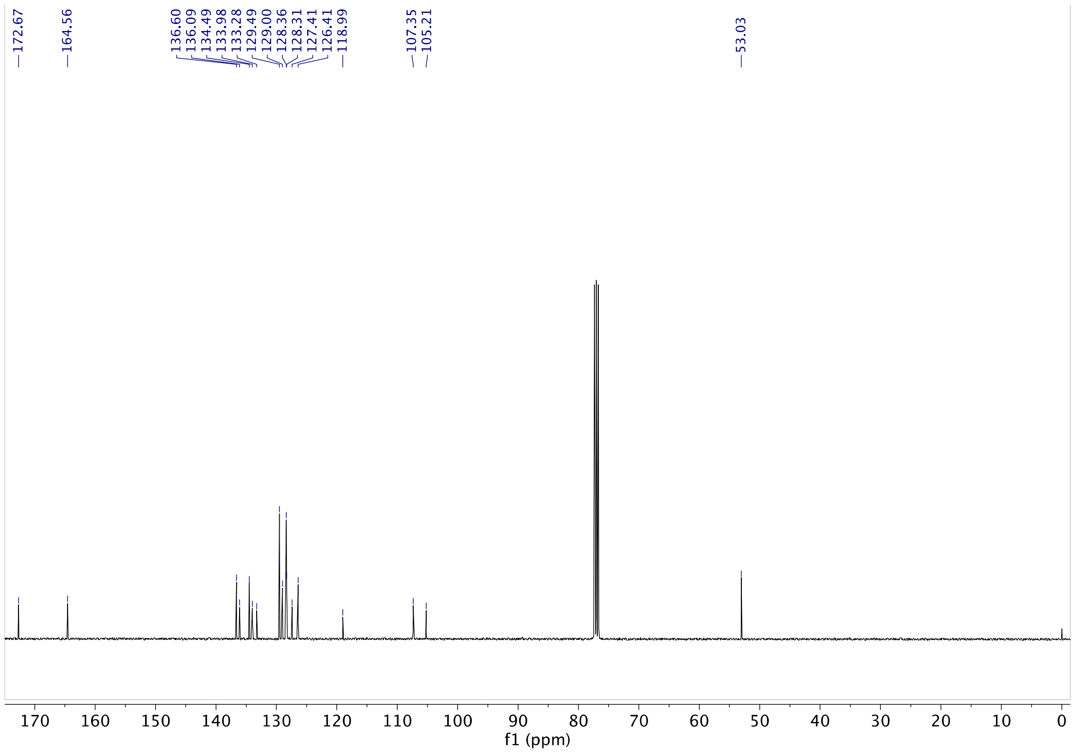

NMR data for methyl 4-(2-fluorophenyl)-3-hydroxy-7-nitro-2-naphthoate (**21**)

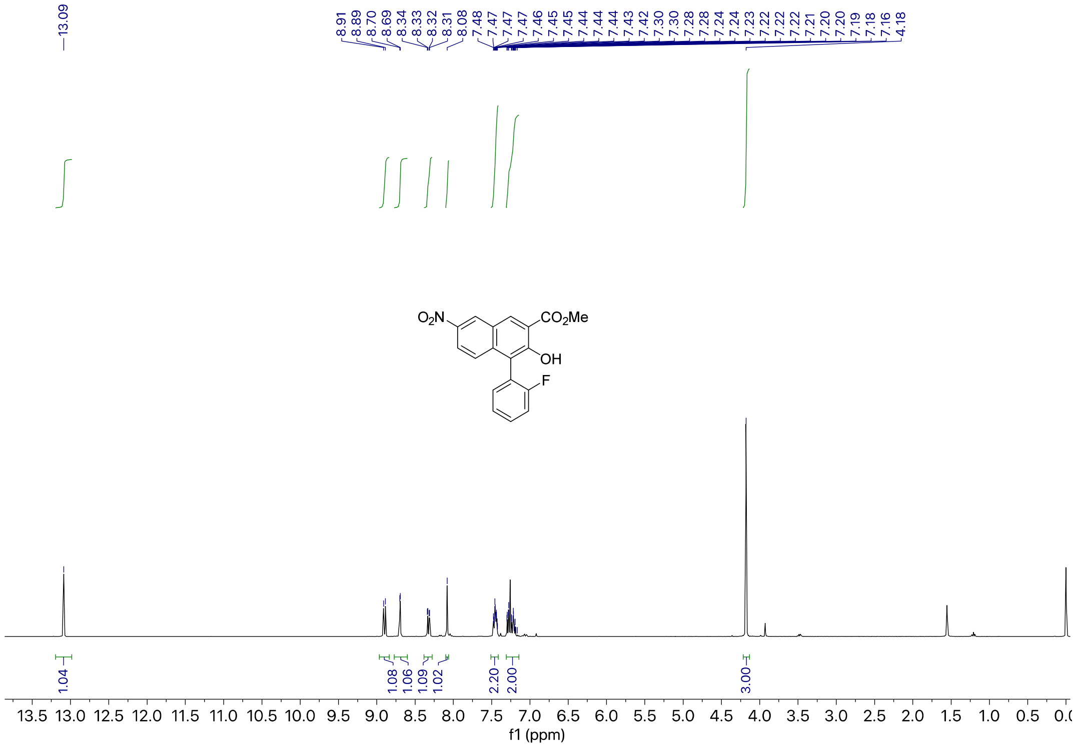

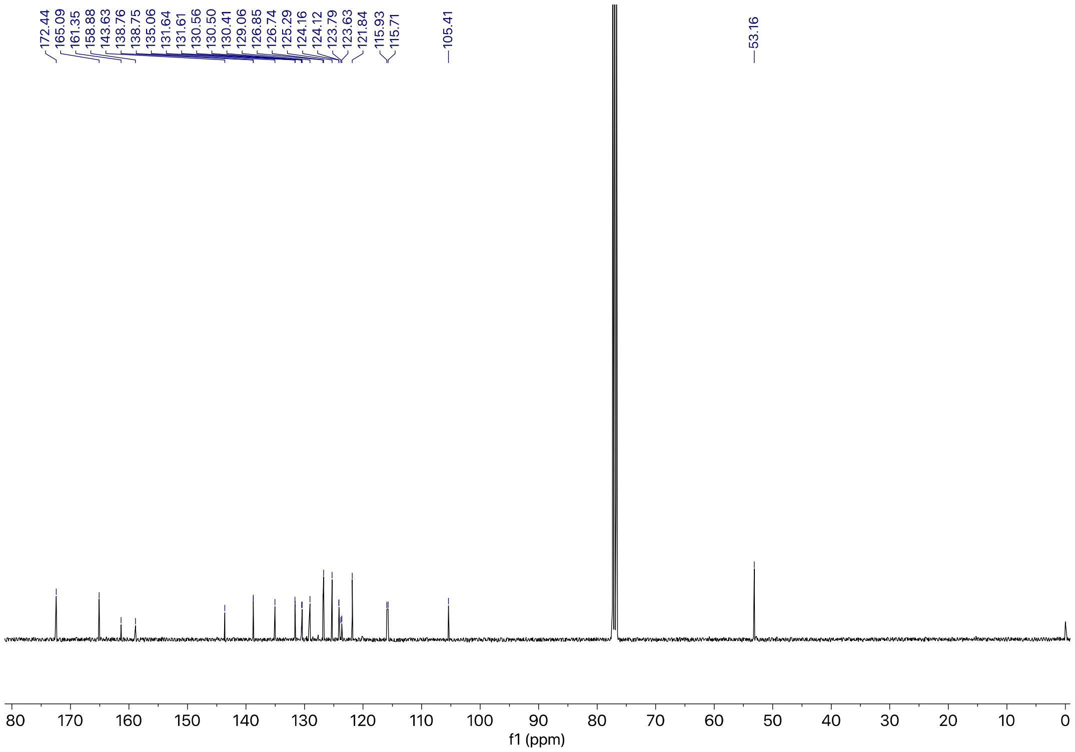

NMR data for methyl 4-(4-chlorophenyl)-3-hydroxy-7-nitro-2-naphthoate (**22**)

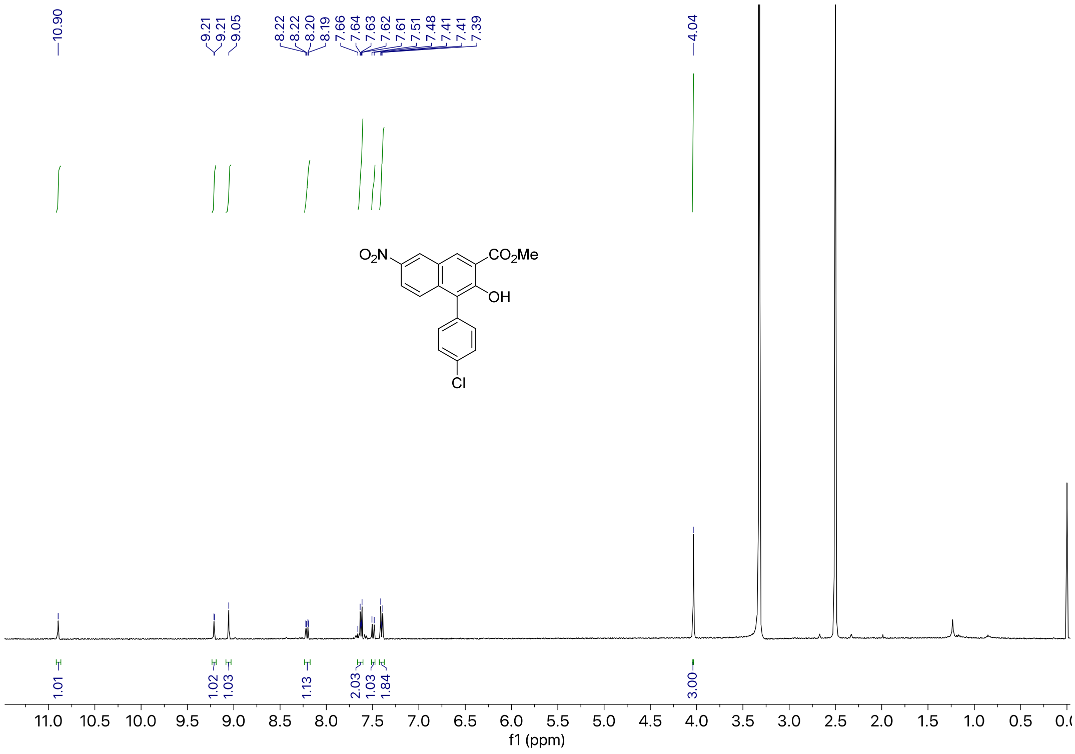

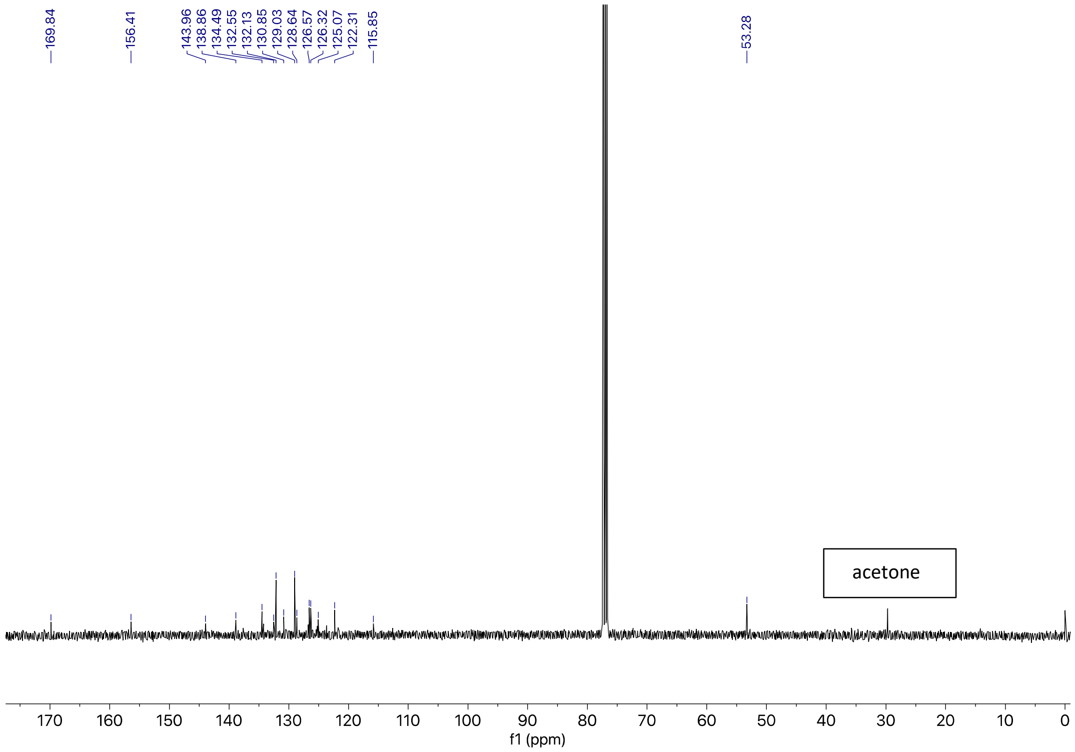

NMR data for methyl 3-hydroxy-4-(4-methylphenyl)-7-nitro-2-naphthoate (**23**)

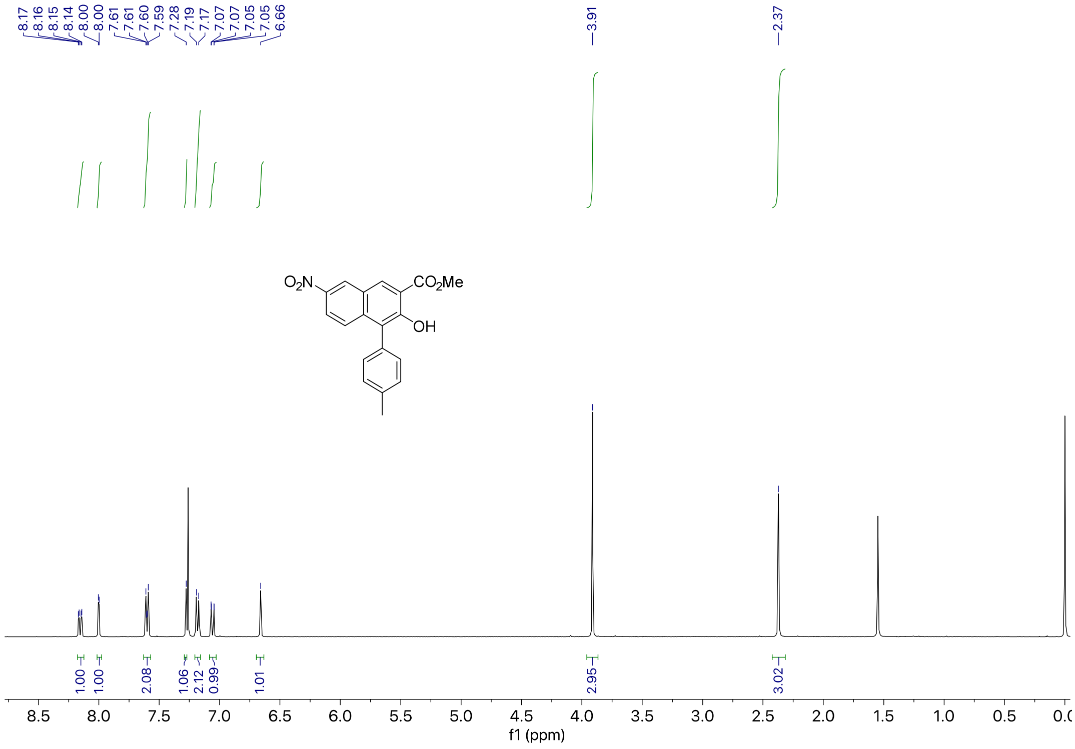

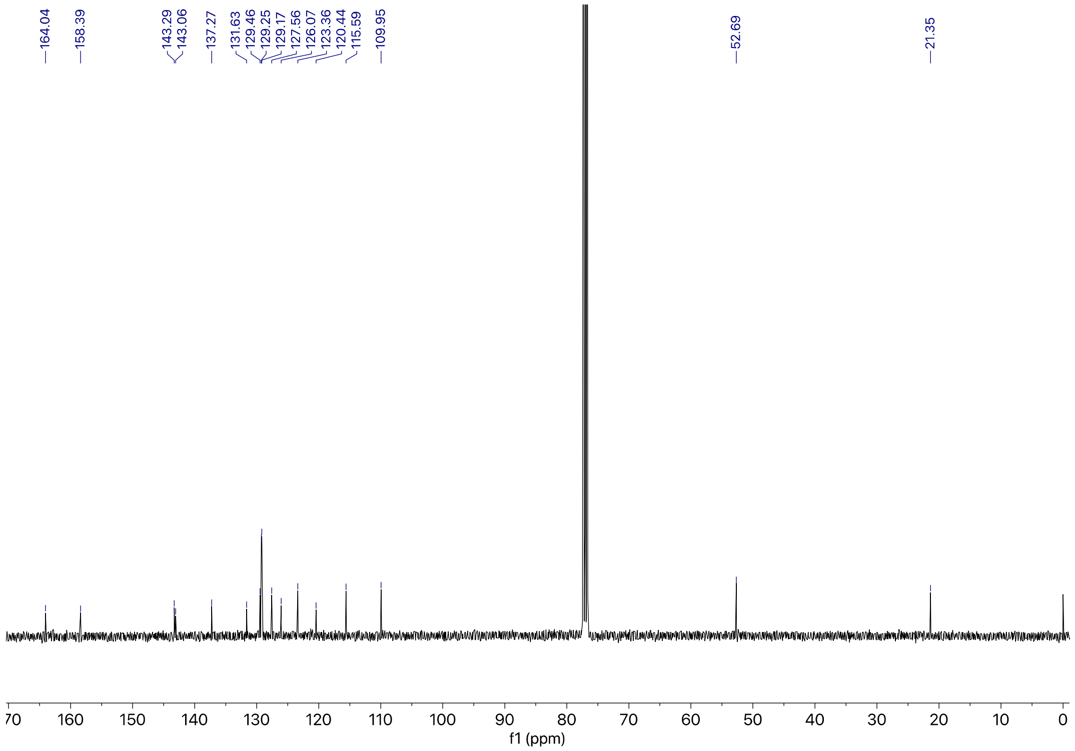

NMR data for methyl 3-hydroxy-4-(4-methoxyphenyl)-7-nitro-2-naphthoate (**24**)

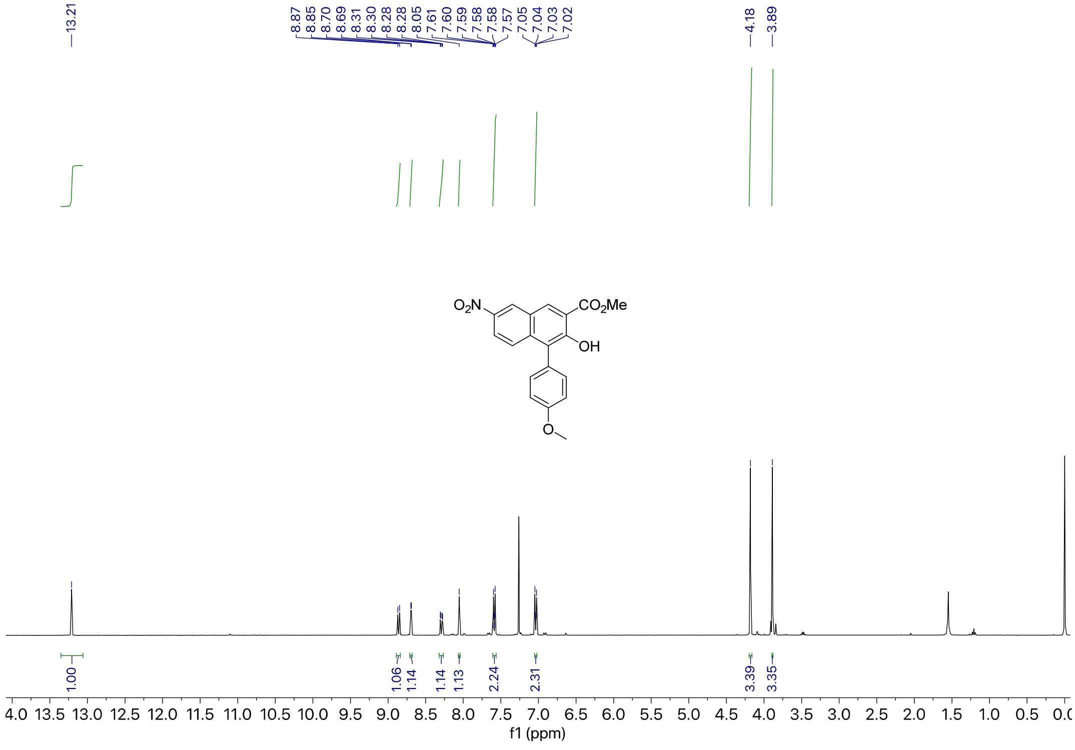

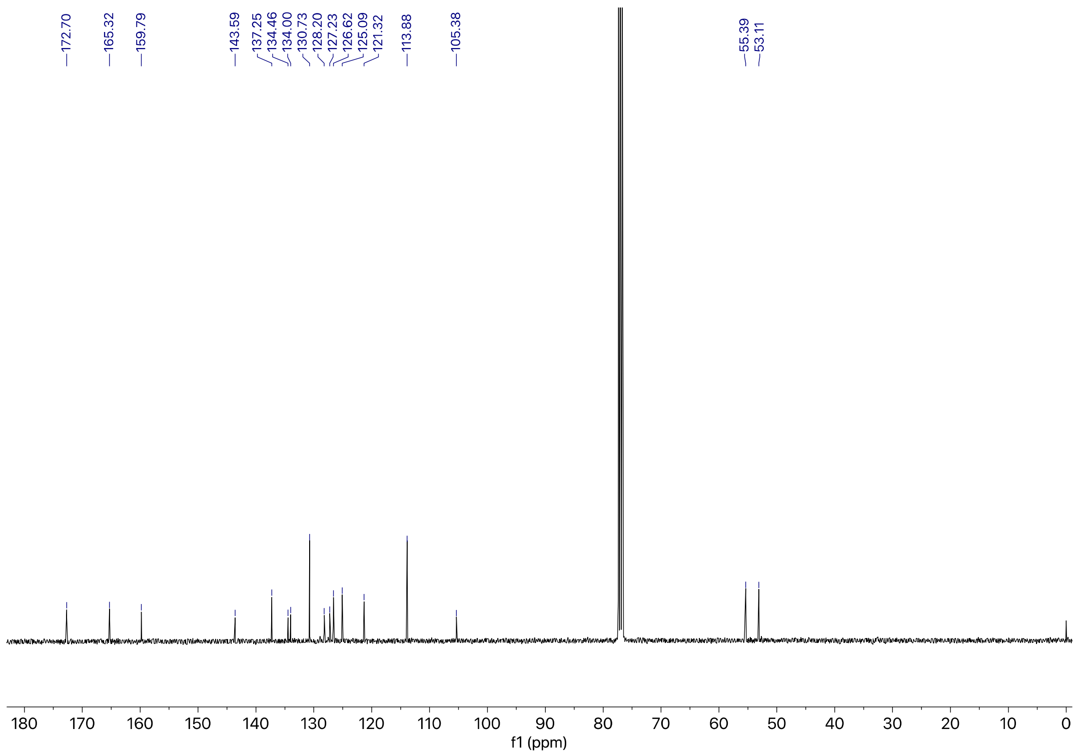

NMR data for methyl 3-hydroxy-7-nitro-4-(3-(trifluoromethyl)phenyl)-2-naphthoate (**25**)

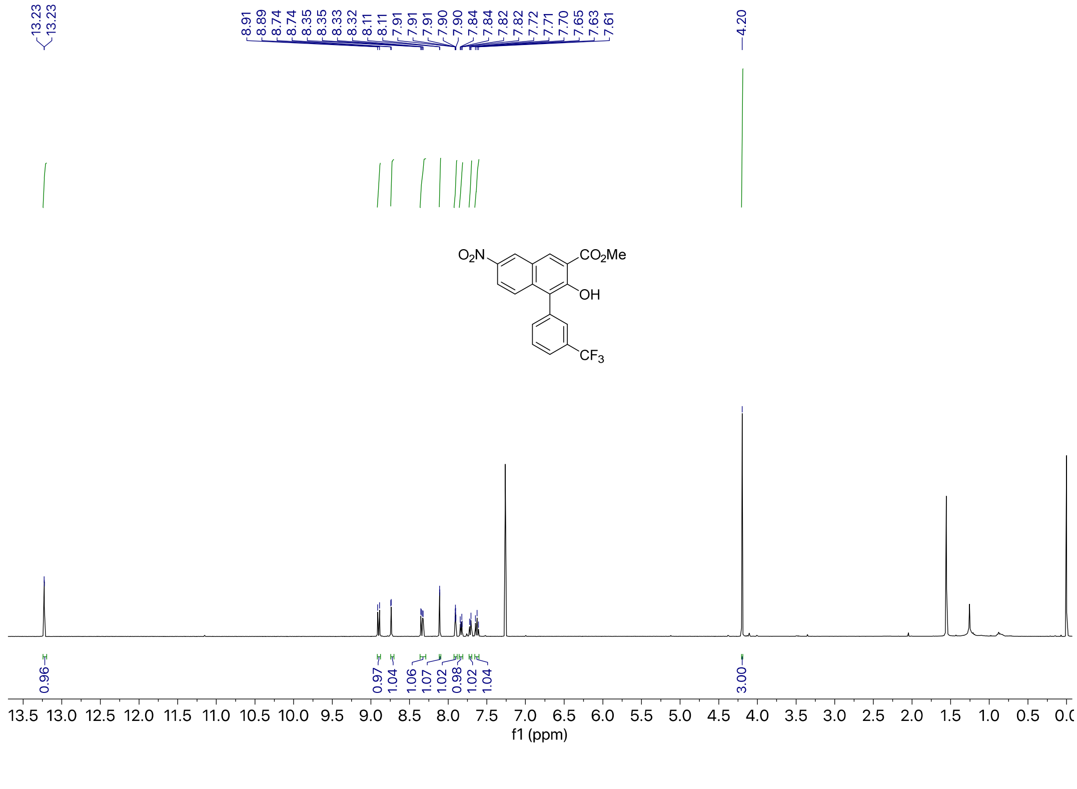

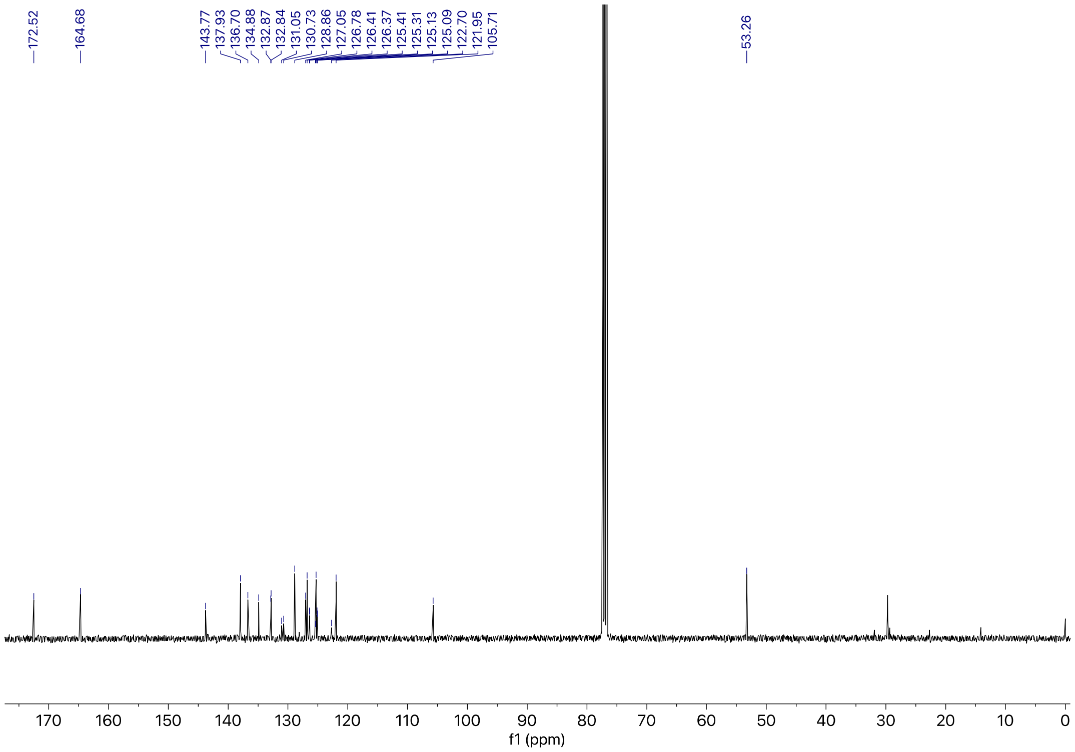

NMR data of methyl 4-(2,5-dimethylphenyl)-3-hydroxy-7-nitro-2-naphthoate (**26**)

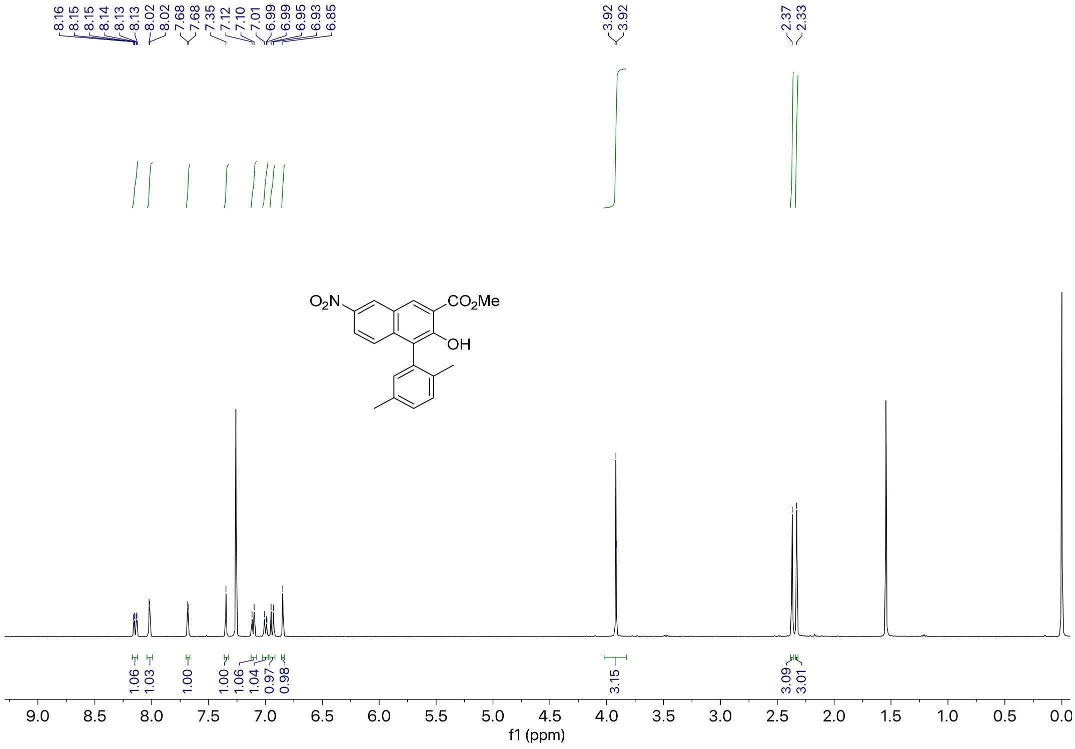

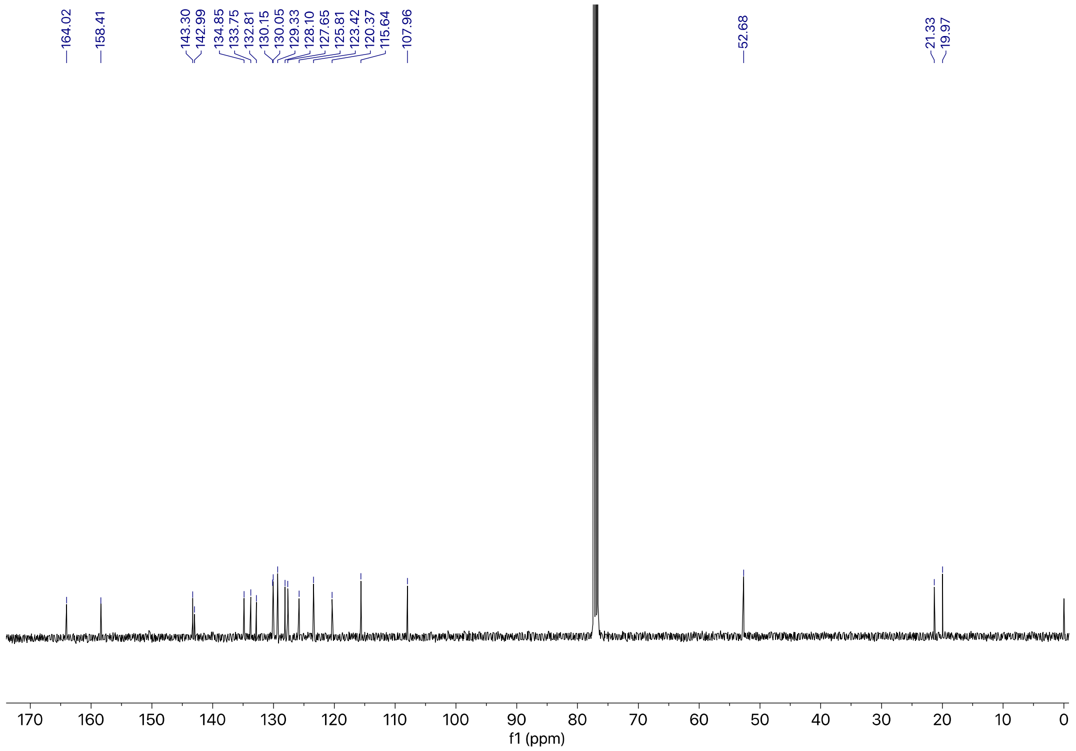

NMR data for 1,3-dibenzoyl-2-hydroxy-6-nitronaphthalene (**27**)

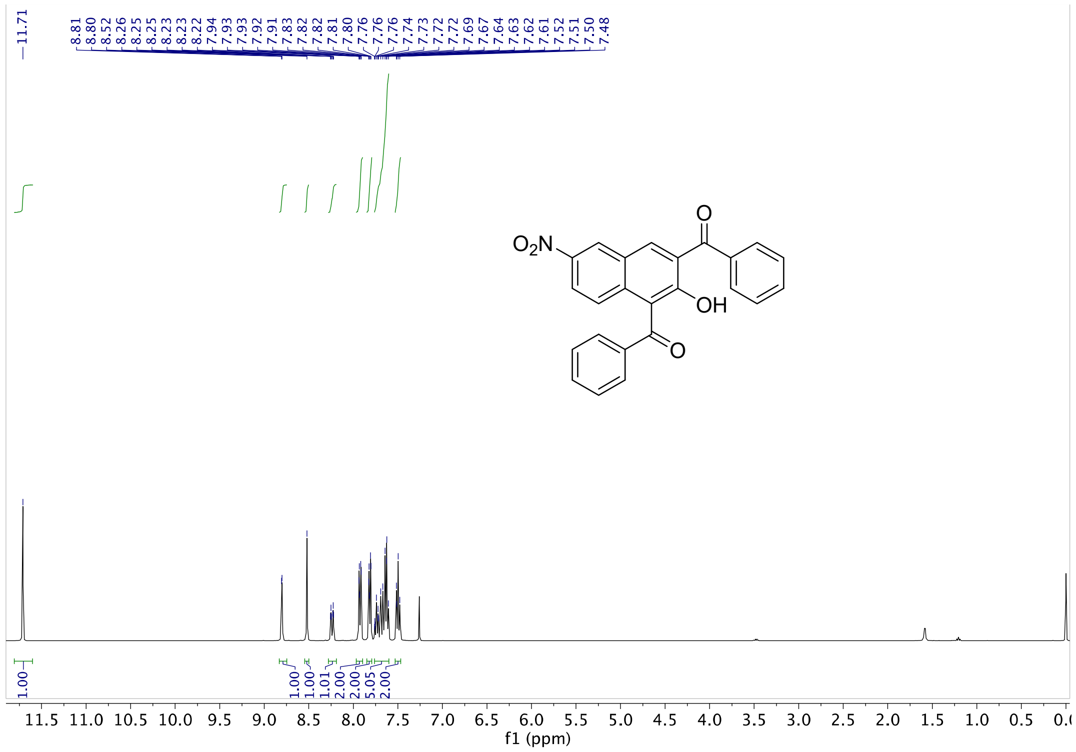

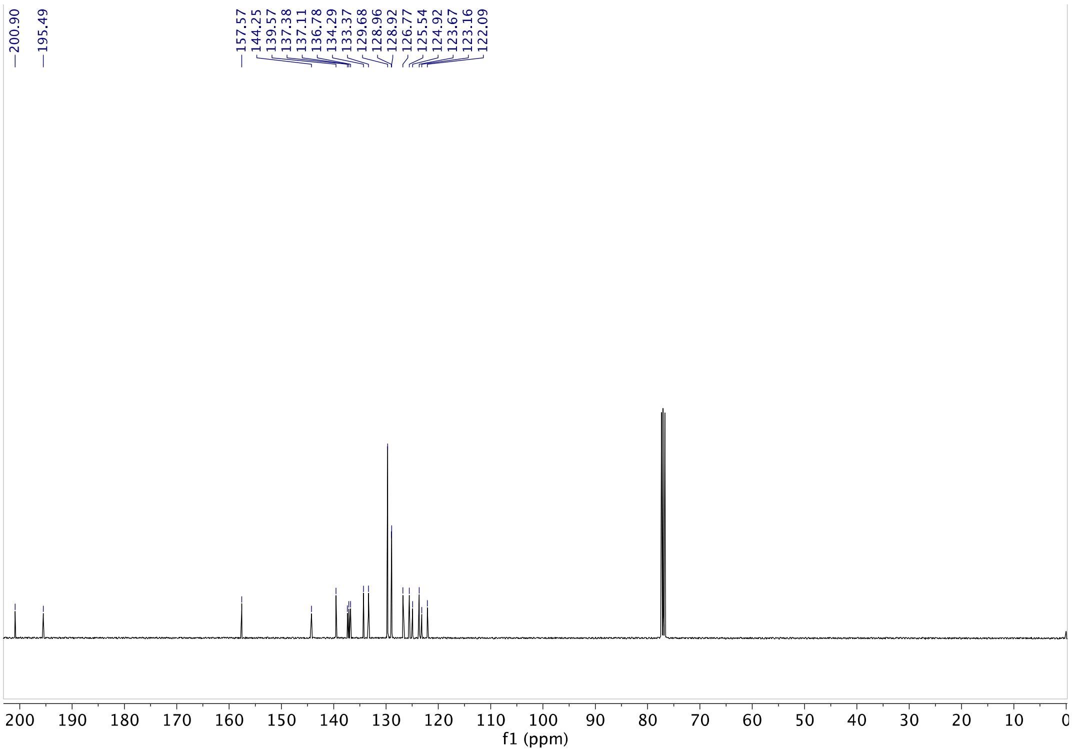

NMR data for 6-nitro-1-phenyl-3-(phenylsulfonyl)naphthalen-2-ol (**28**)

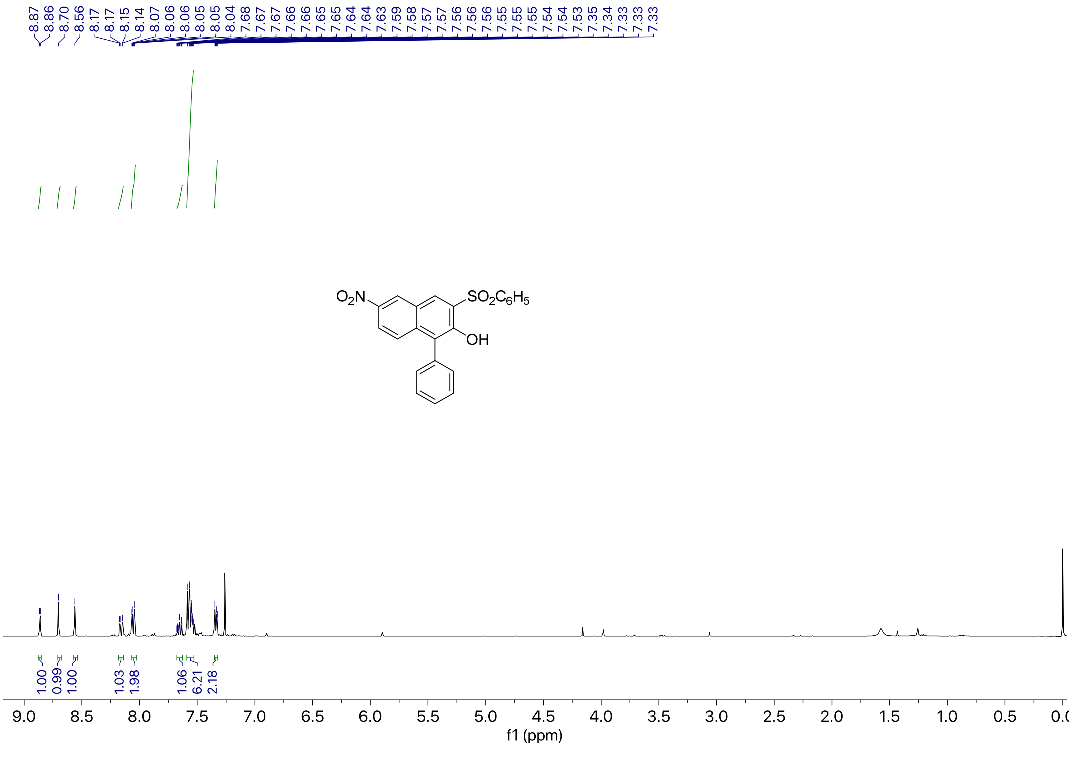

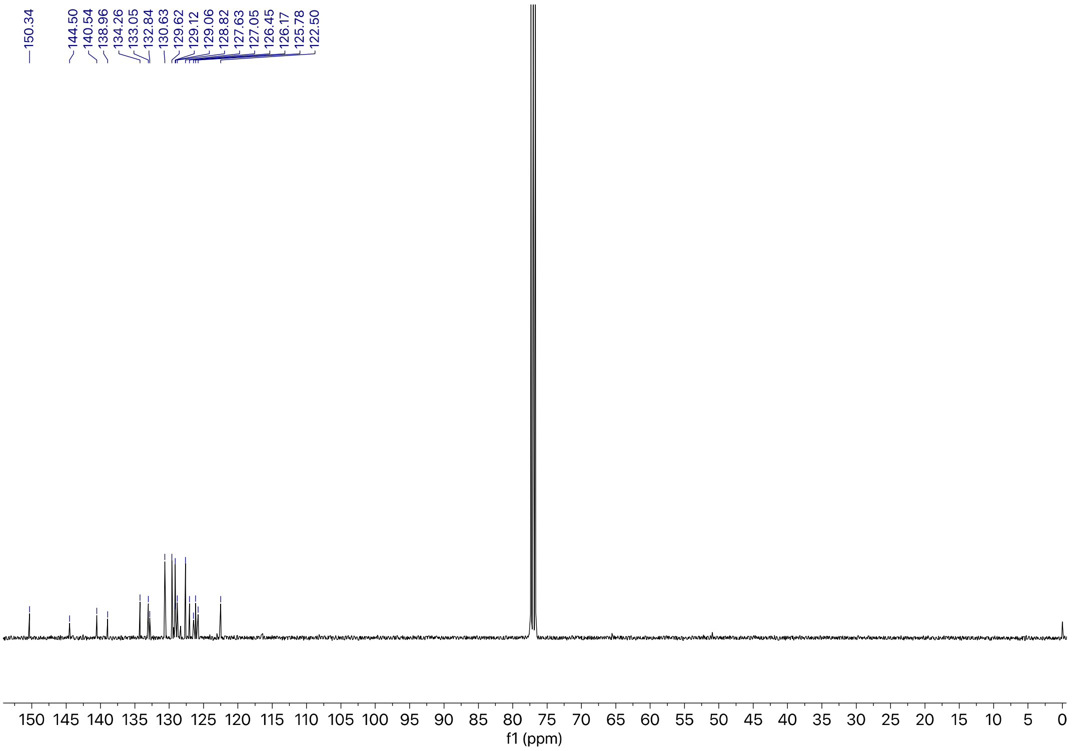

NMR data for 5-nitro-2-(phenylsulfonyl)benzaldehyde (**29**)

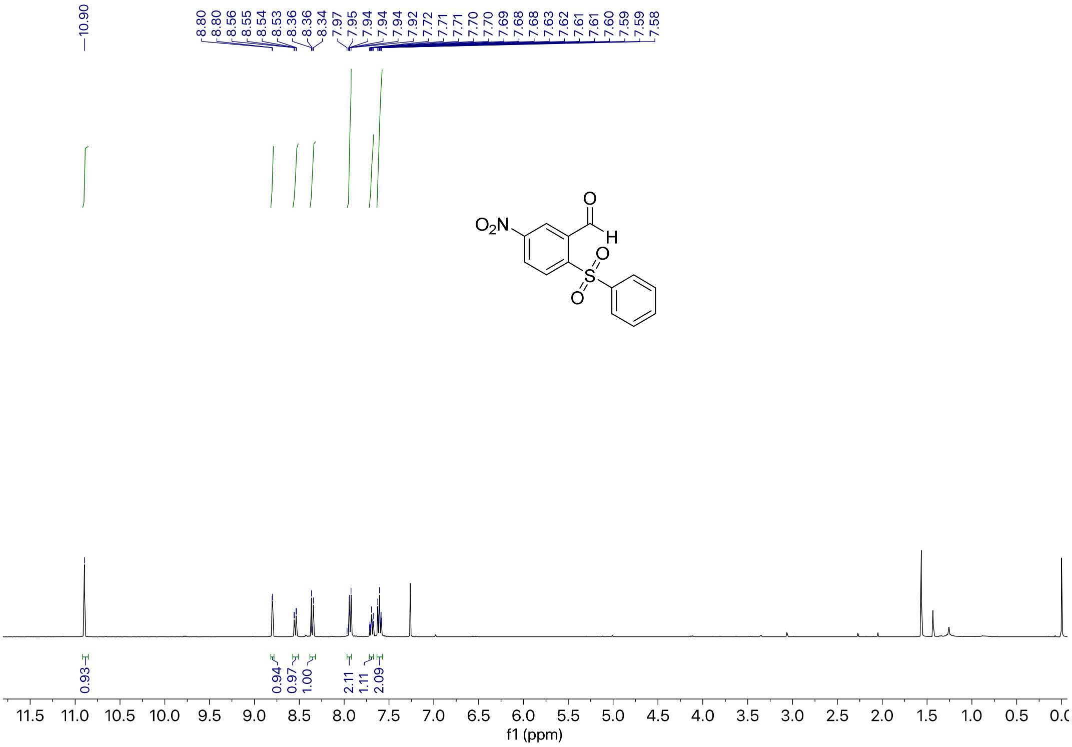

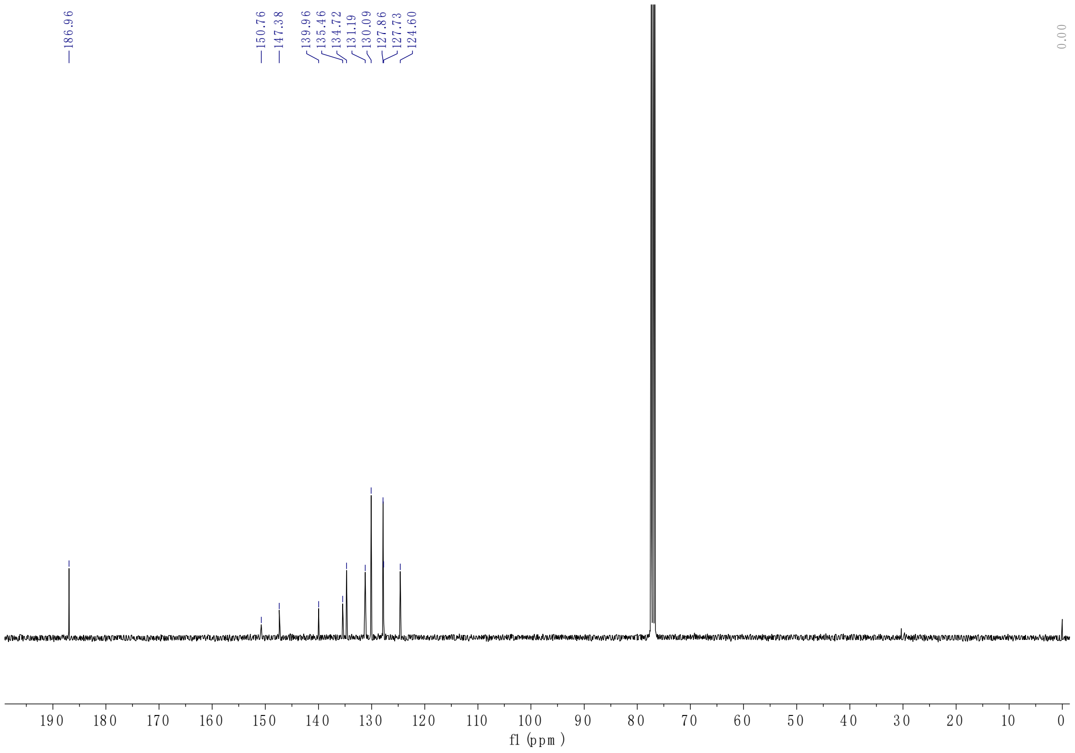

NMR data of dimethyl 6-cyano-2-hydroxynaphthalene-1,3-dicarboxylate (**30**)

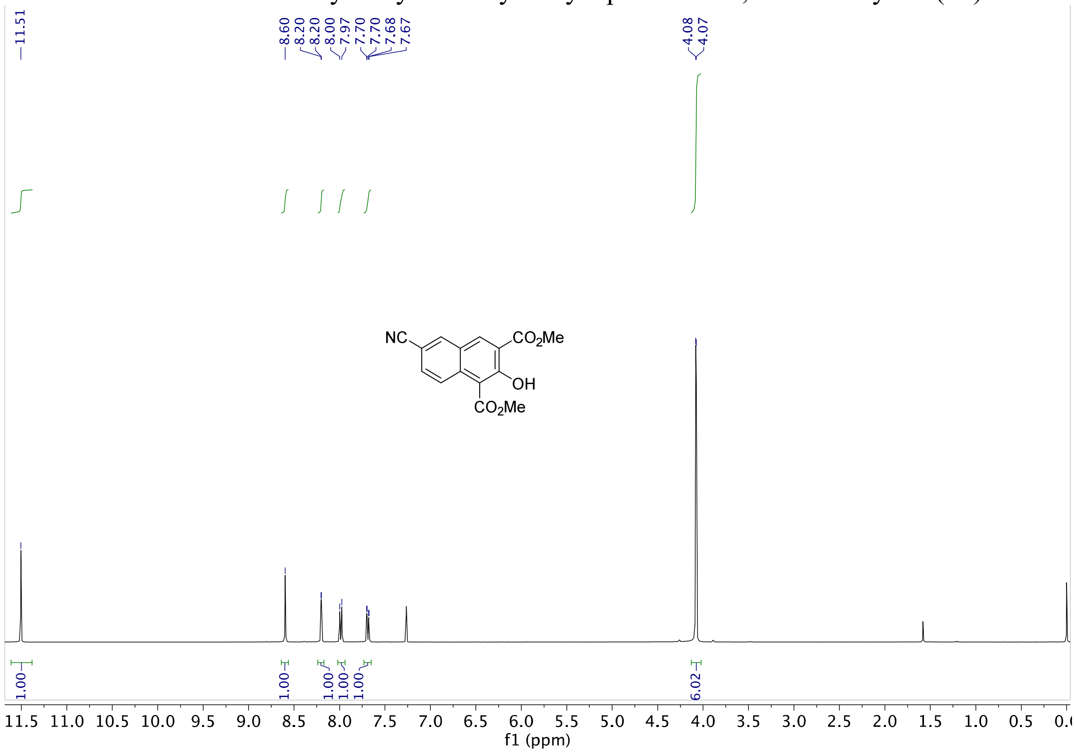

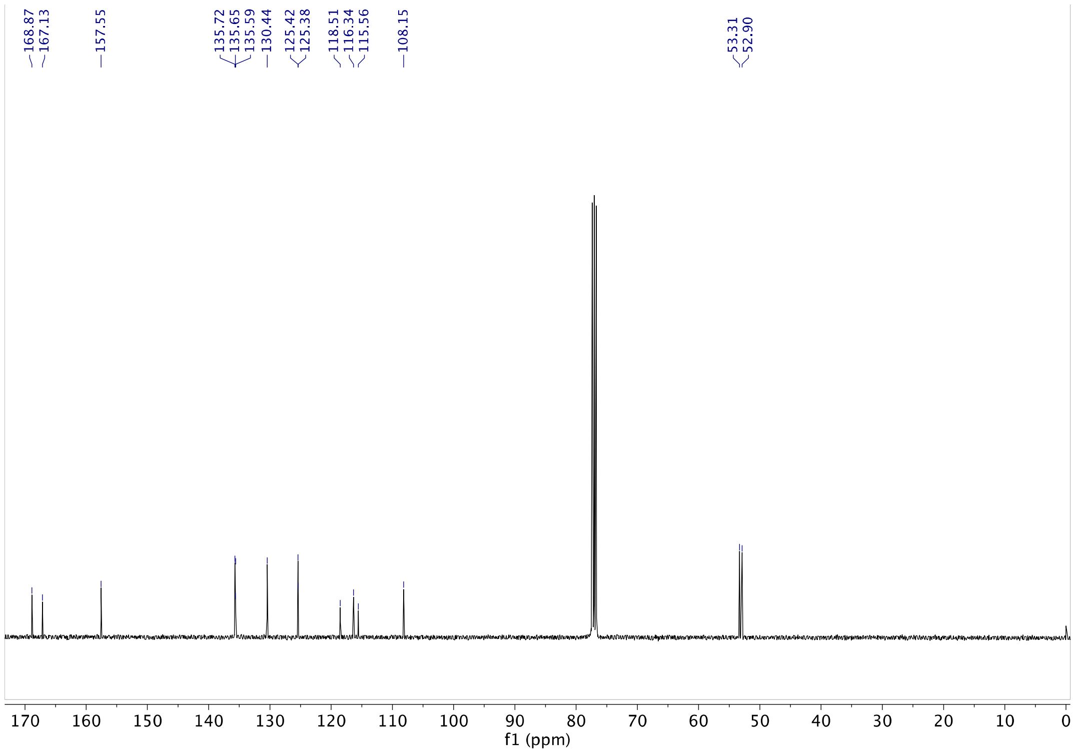

NMR data for methyl 7-cyano-3-hydroxy-4-phenyl-2-naphthoate (**31**)

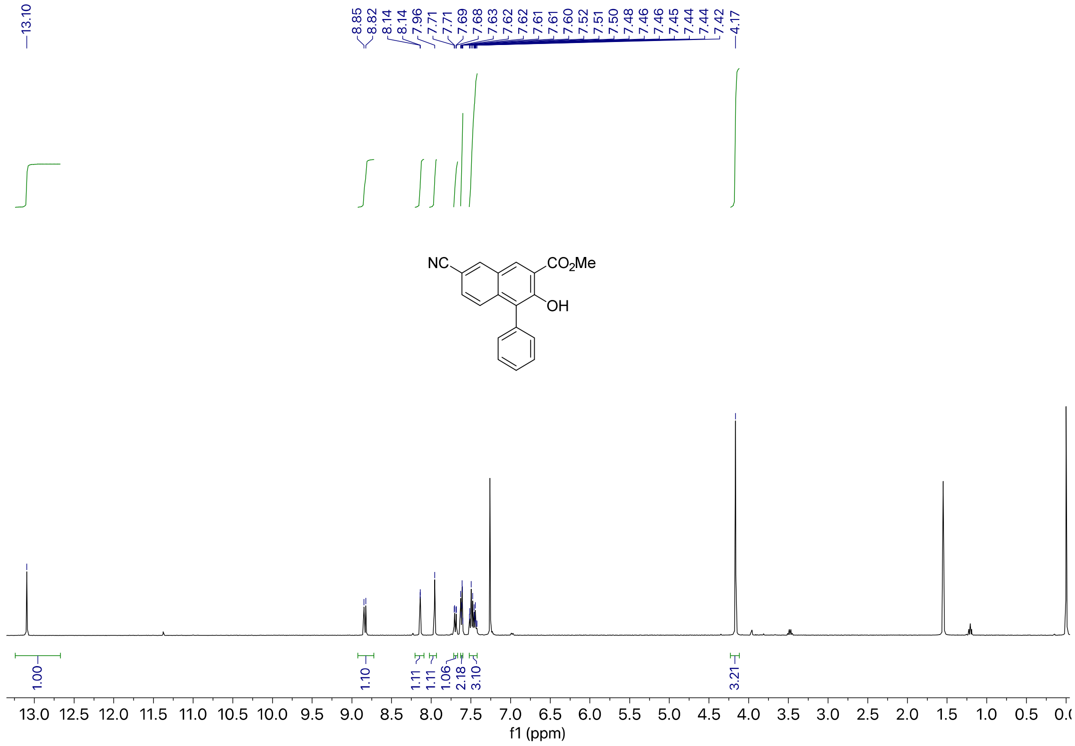

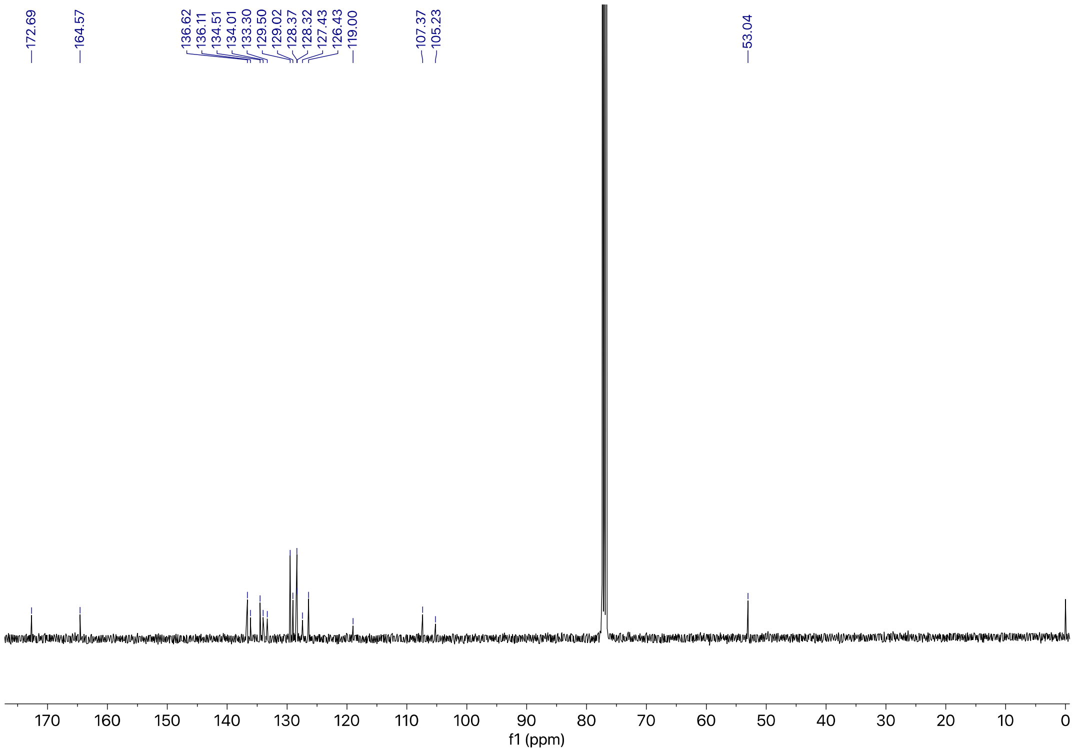

NMR data for methyl 7-cyano-4-(2-fluorophenyl)-3-hydroxy-2-naphthoate (**32**)

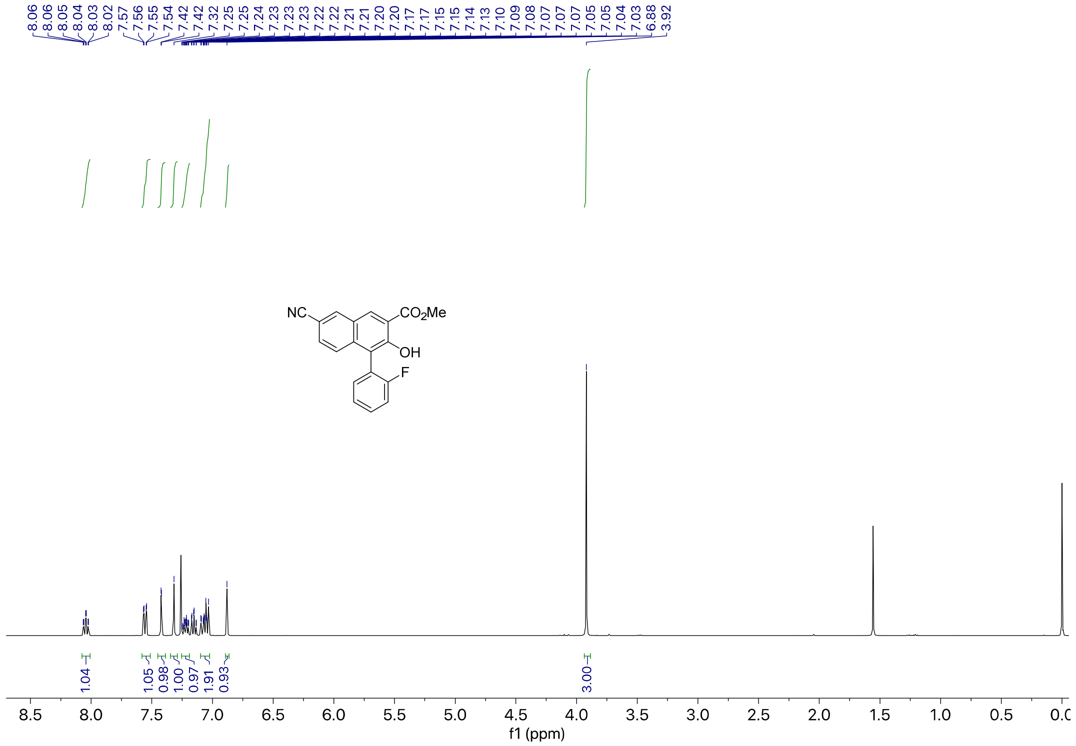

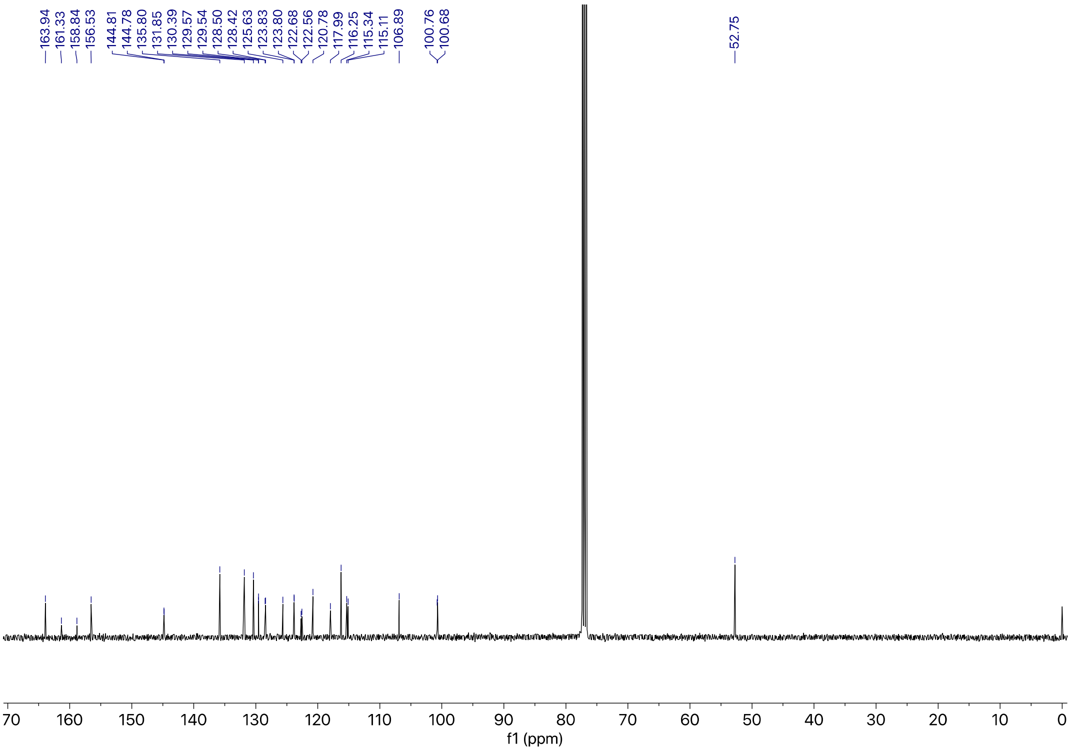

NMR data for methyl 4-(4-chlorophenyl)-7-cyano-3-hydroxy-2-naphthoate (**33**)

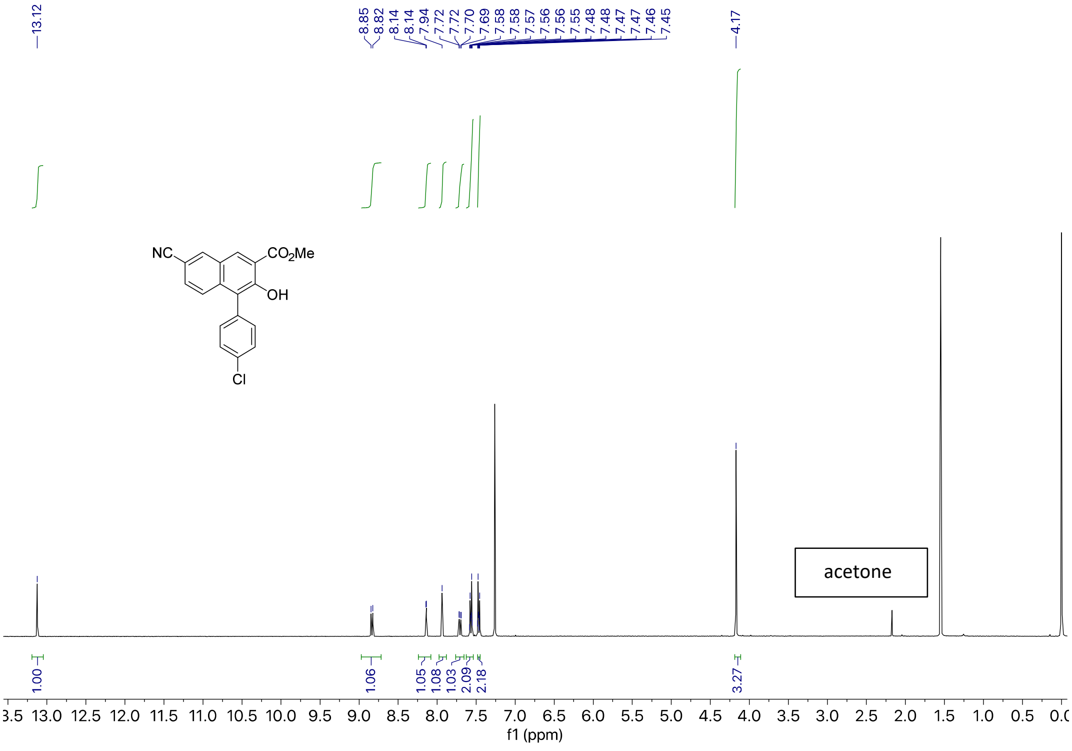

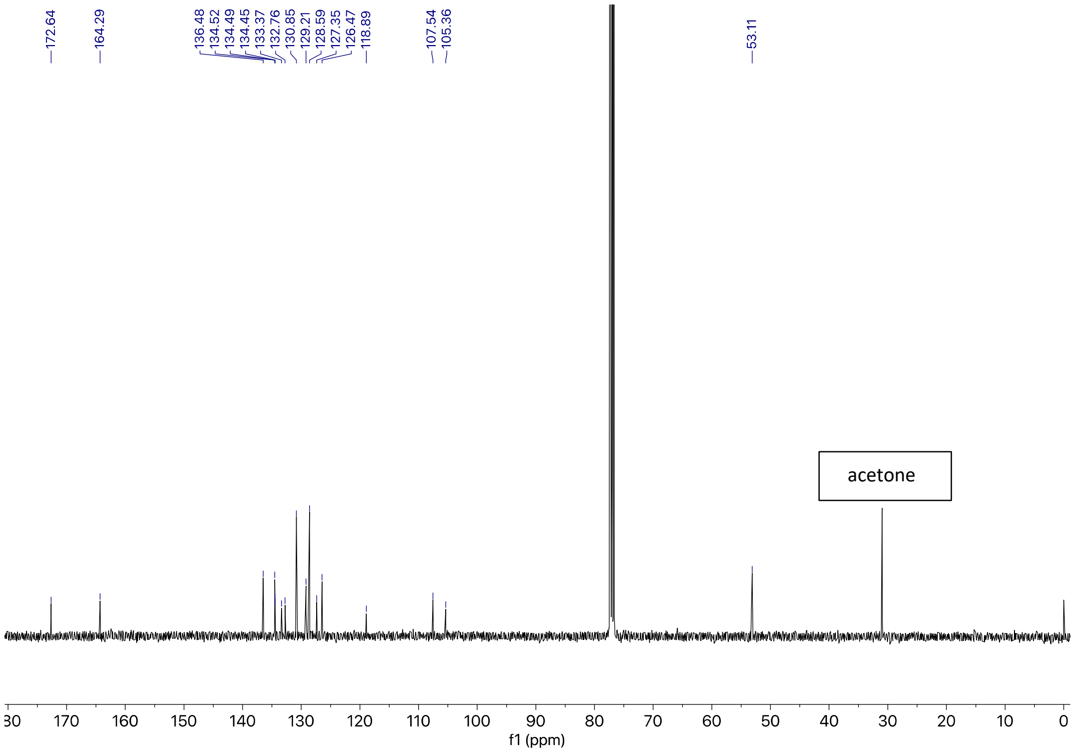

NMR data for methyl 7-cyano-3-hydroxy-4-(4-methoxyphenyl)-2-naphthoate (**34**)

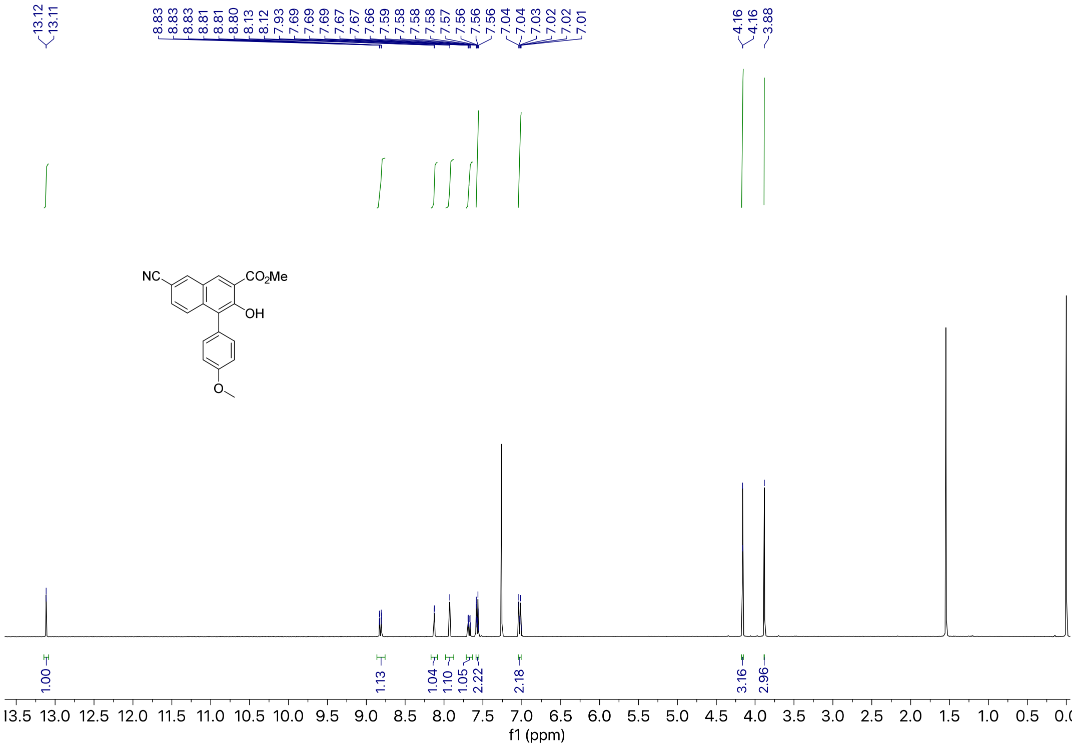

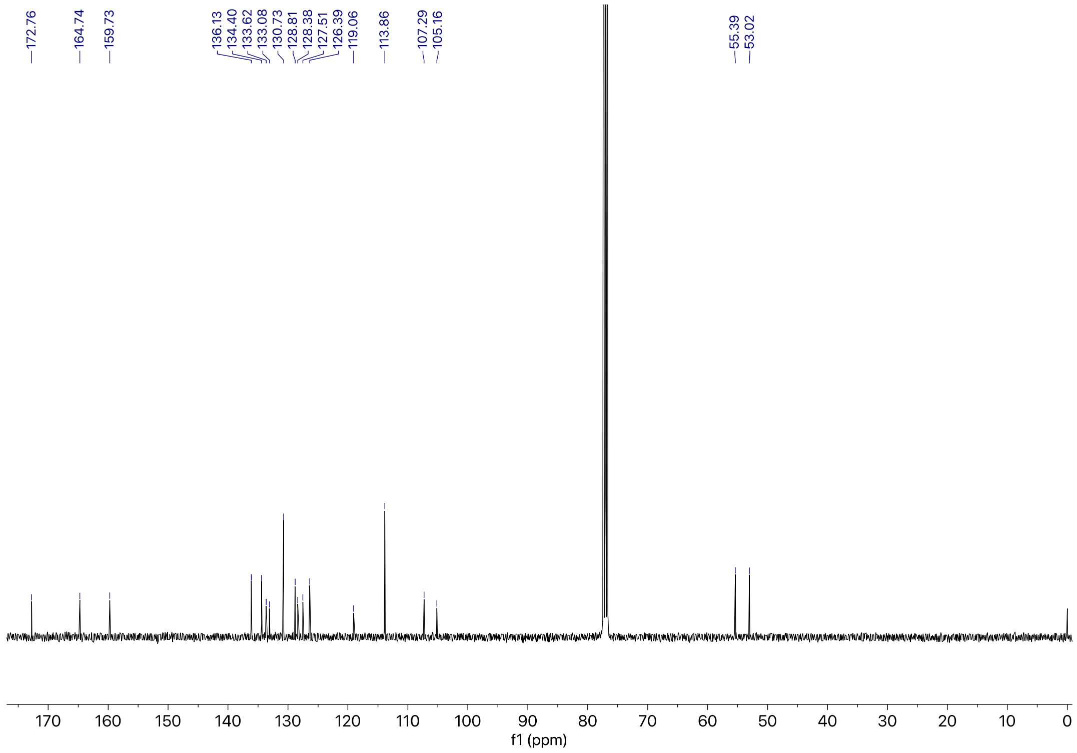

NMR data for methyl 7-cyano-4-(2,5-dimethylphenyl)-3-hydroxy-2-naphthoate (**35**)

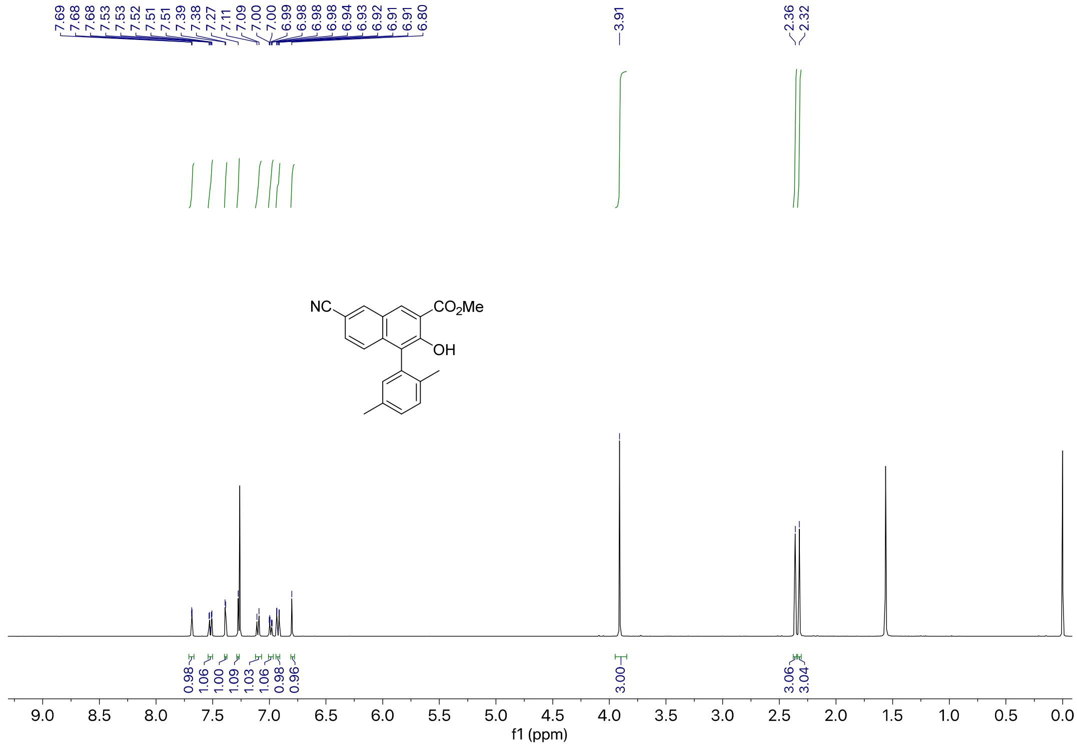

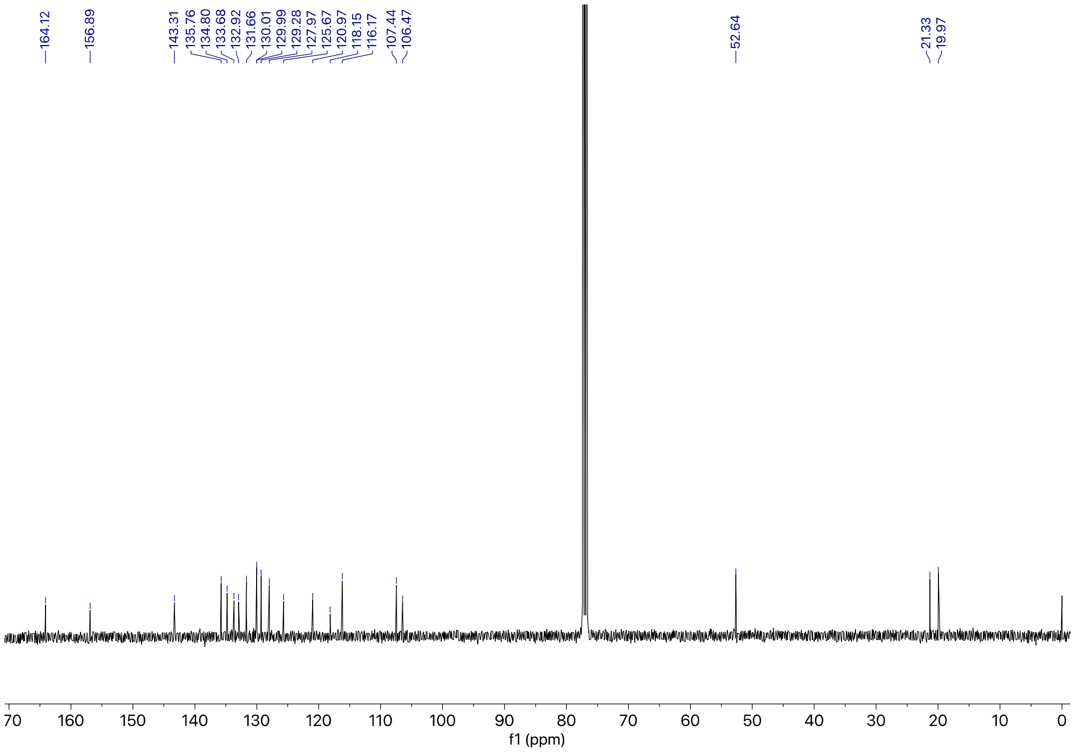

NMR data for 5,7-dibenzoyl-6-hydroxy-2-naphthonitrile (**36**)

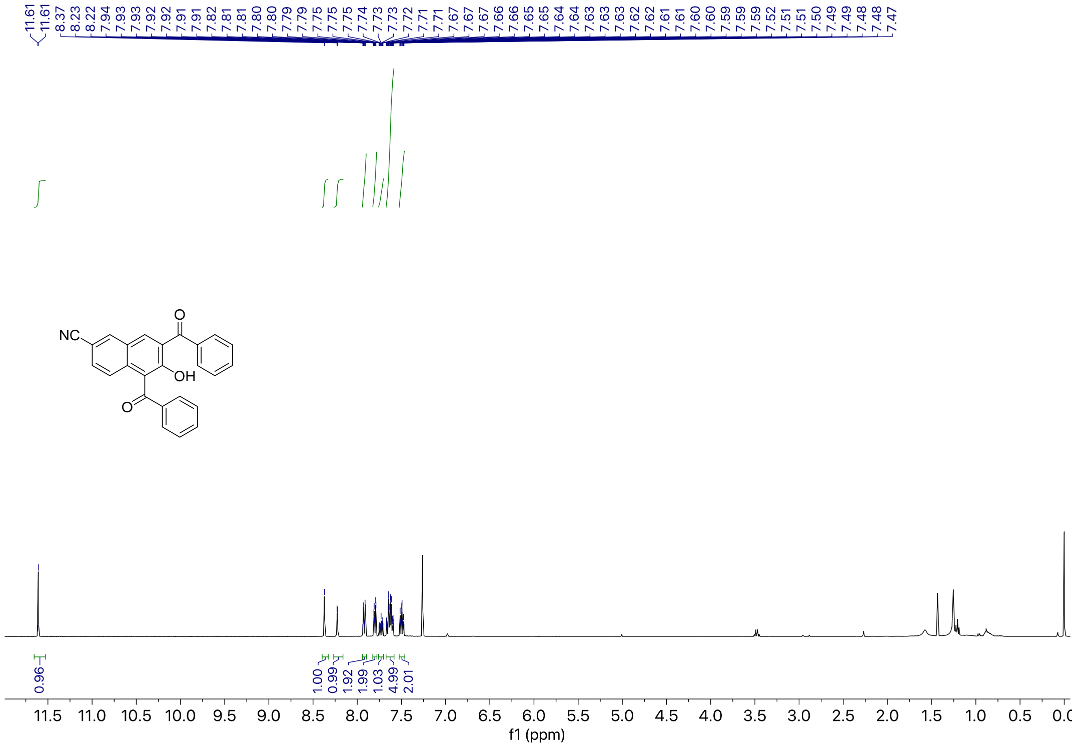

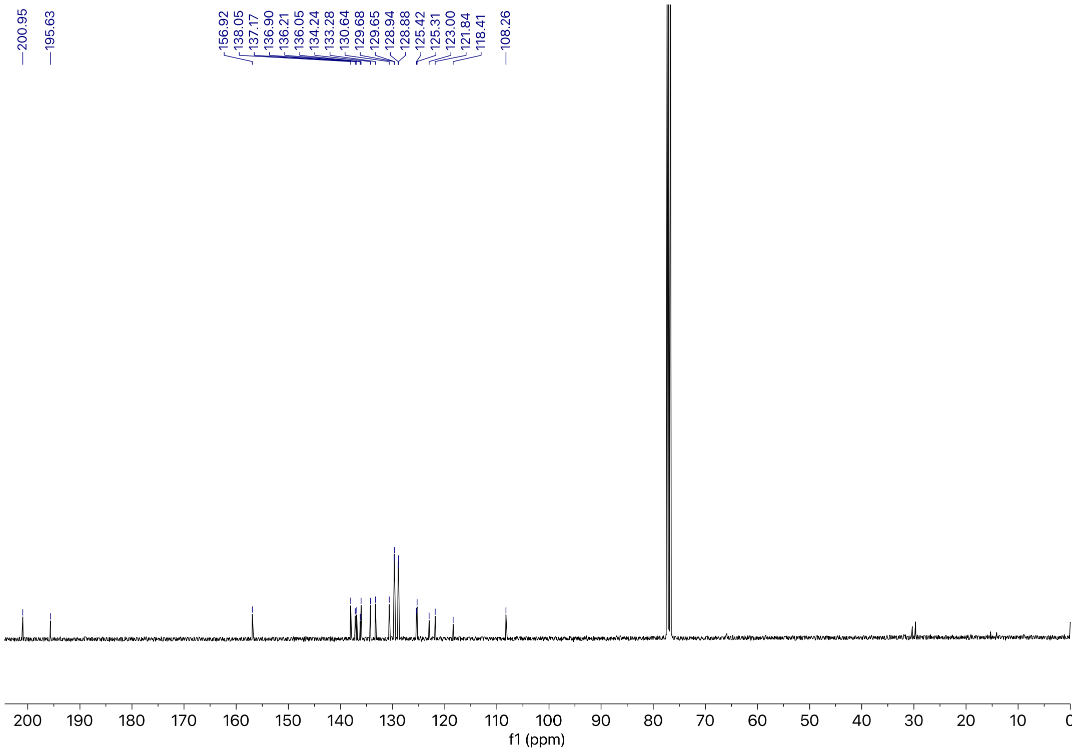

NMR data for ethyl (*E*)-3-(2-fluoro-5-(trifluoromethyl)phenyl)acrylate (**37**)

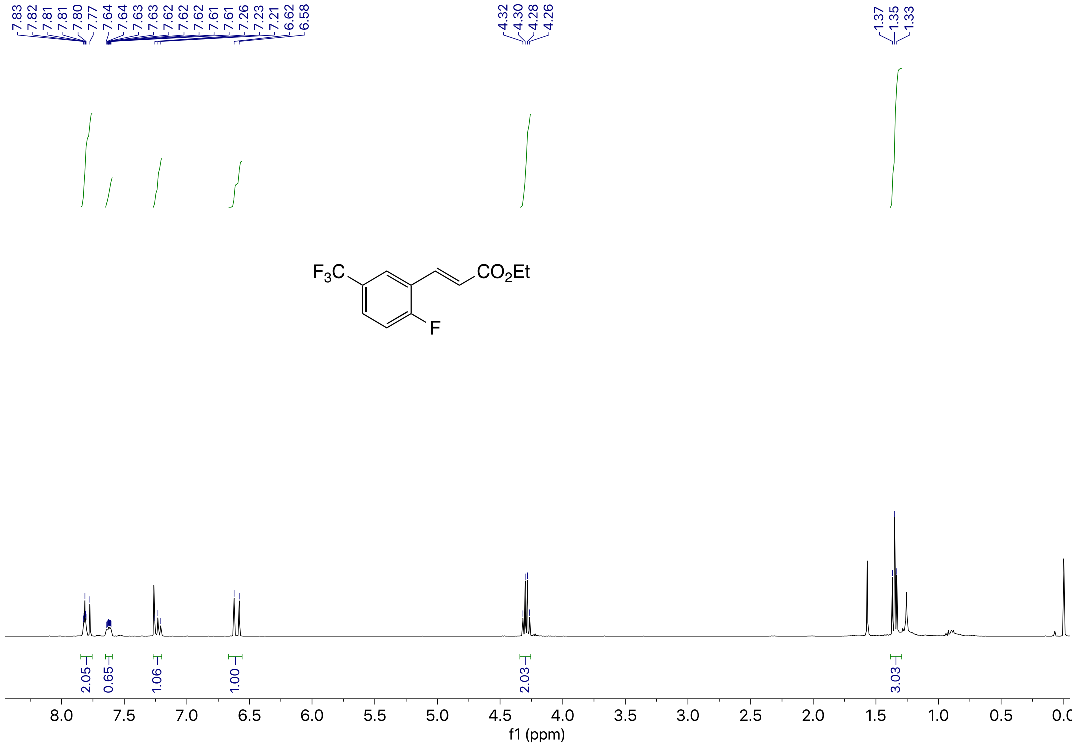

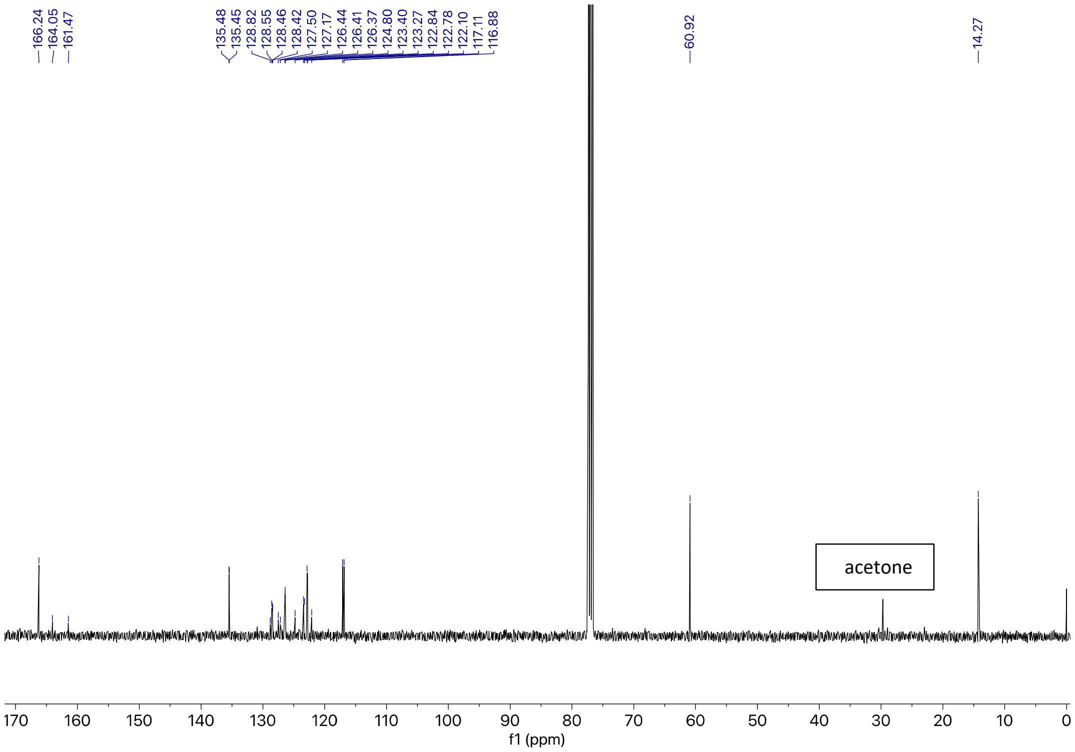

NMR data for methyl 3-hydroxy-4-(p-tolyl)-7-(trifluoromethyl)-2-naphthoate (**38**)

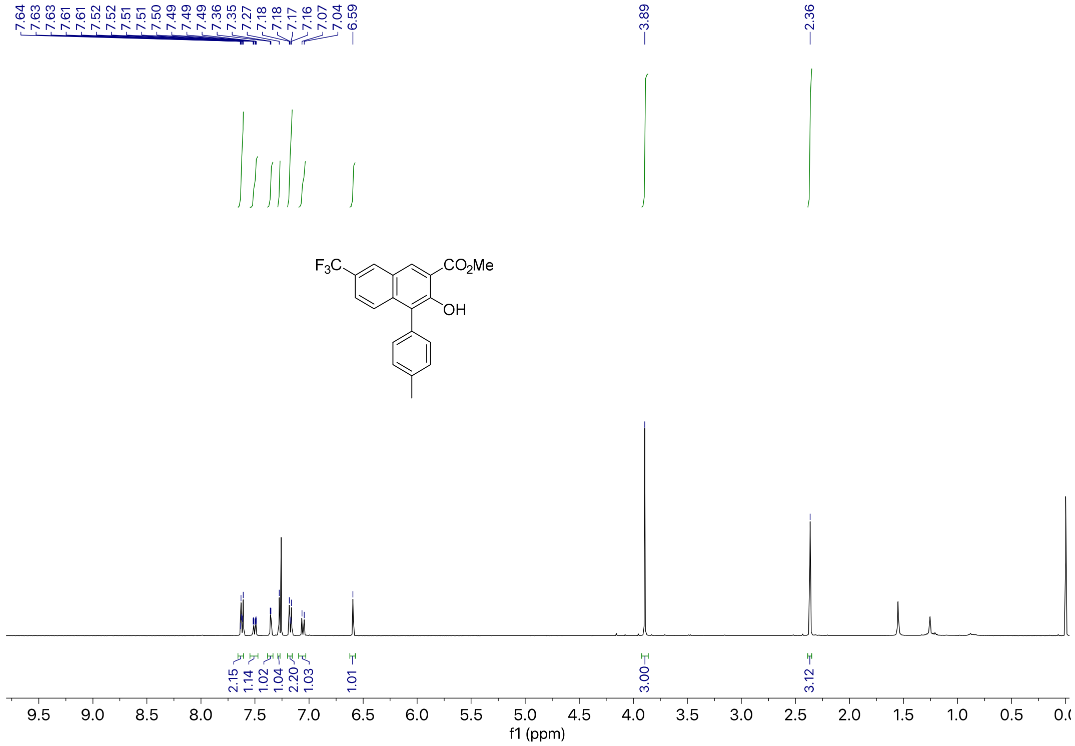

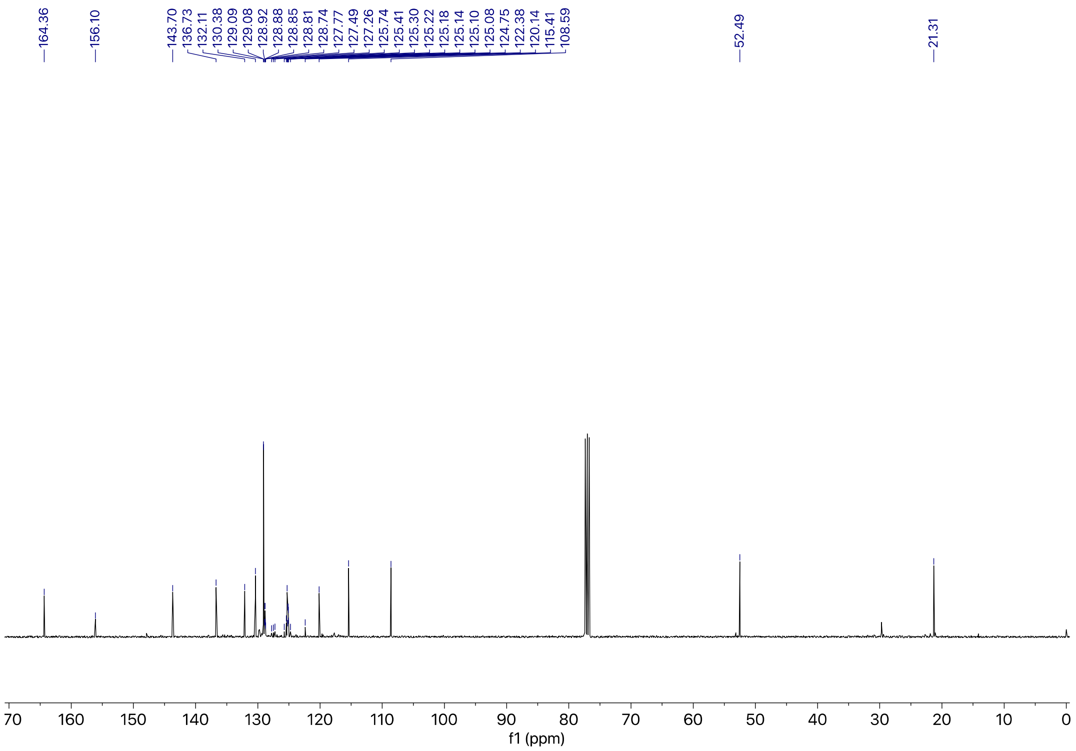

NMR data for methyl 3-hydroxy-4-(4-methoxyphenyl)-7-(trifluoromethyl)-2-naphthoate (**39**)

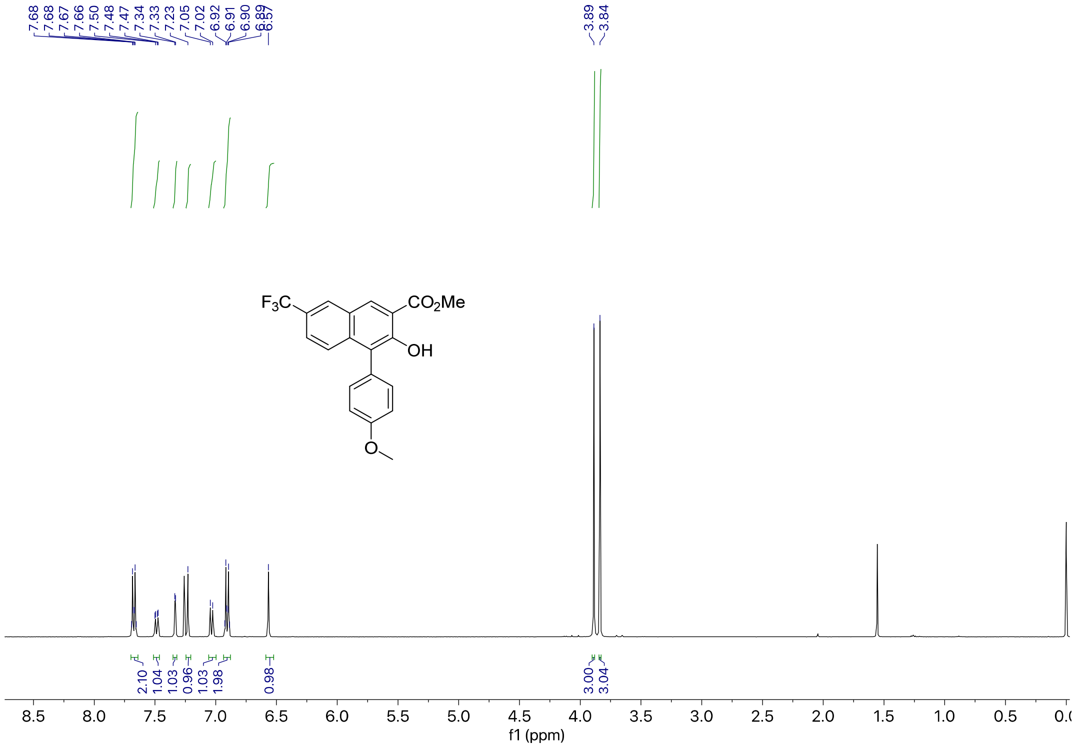

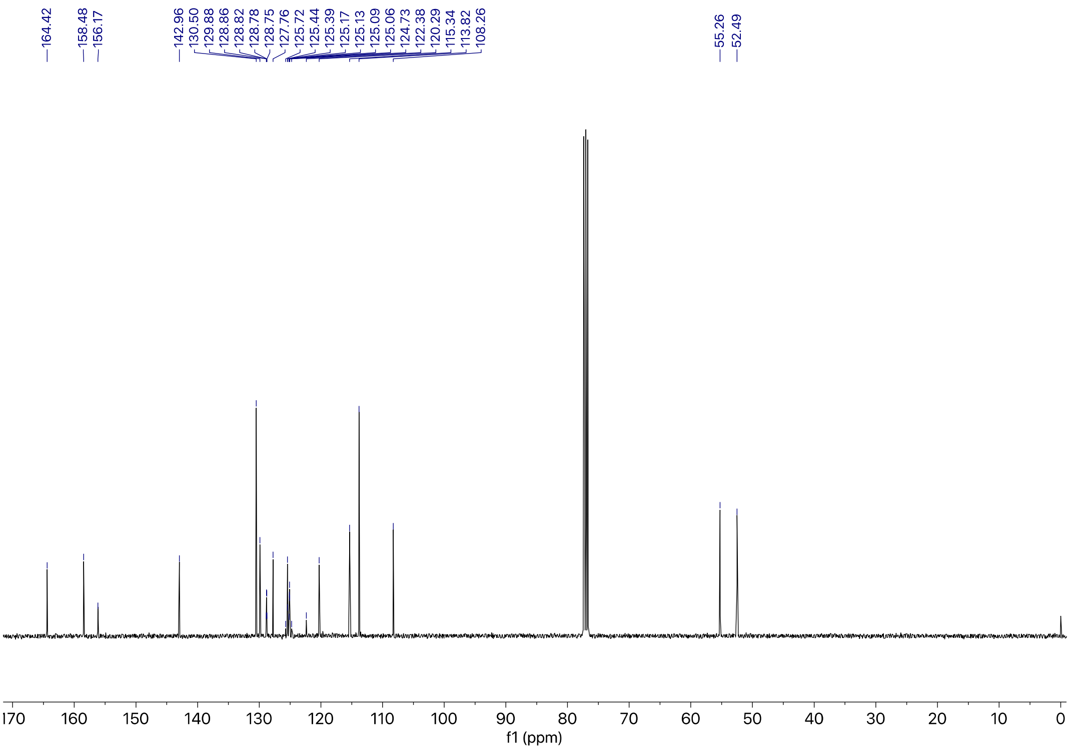

NMR data for ethyl (*E*)-3-(2-fluoropyridin-3-yl)acrylate (**40**)

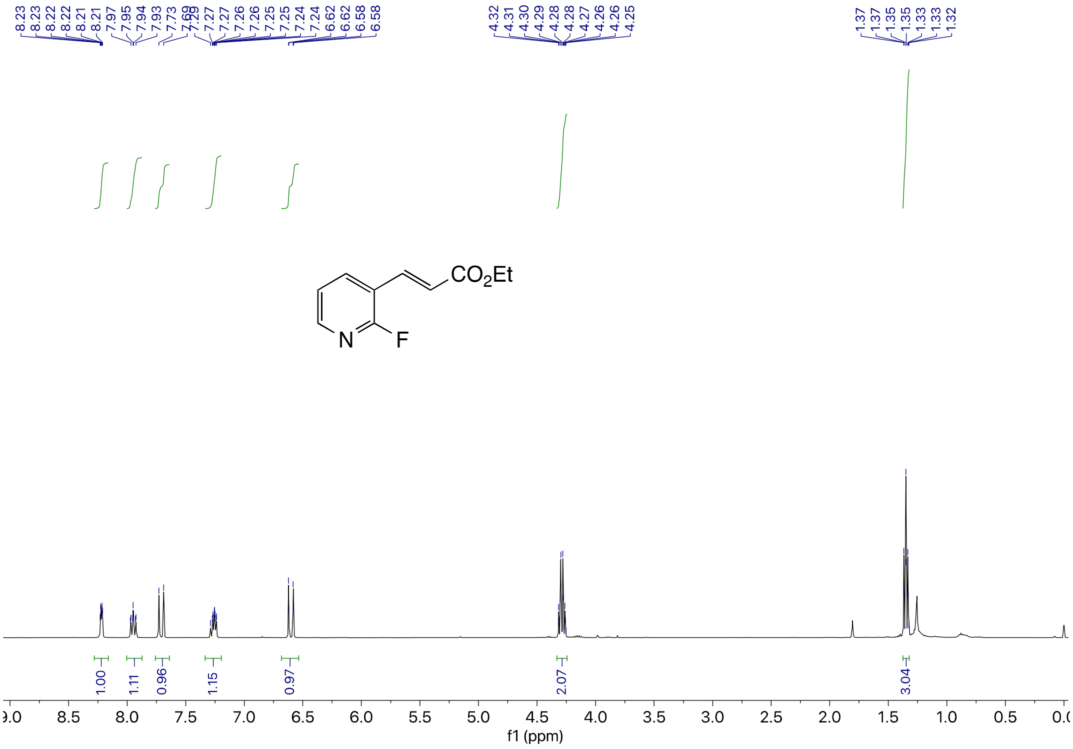

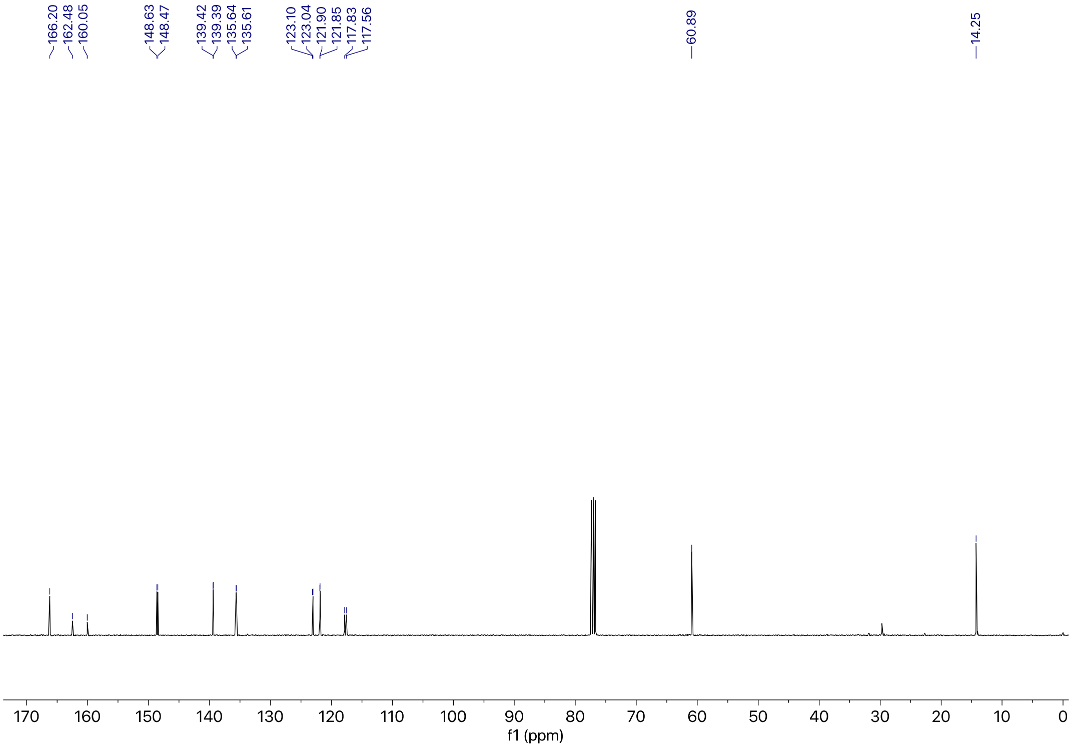

NMR data for methyl 7-hydroxy-8-phenylquinoline-6-carboxylate (**41**)

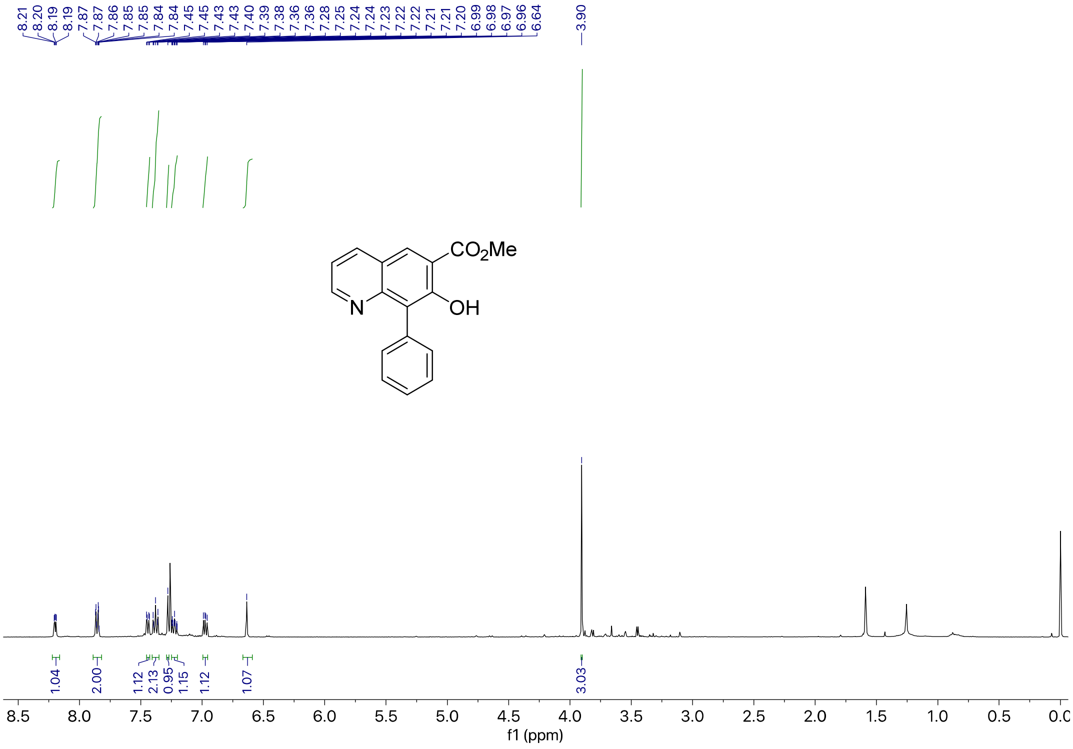

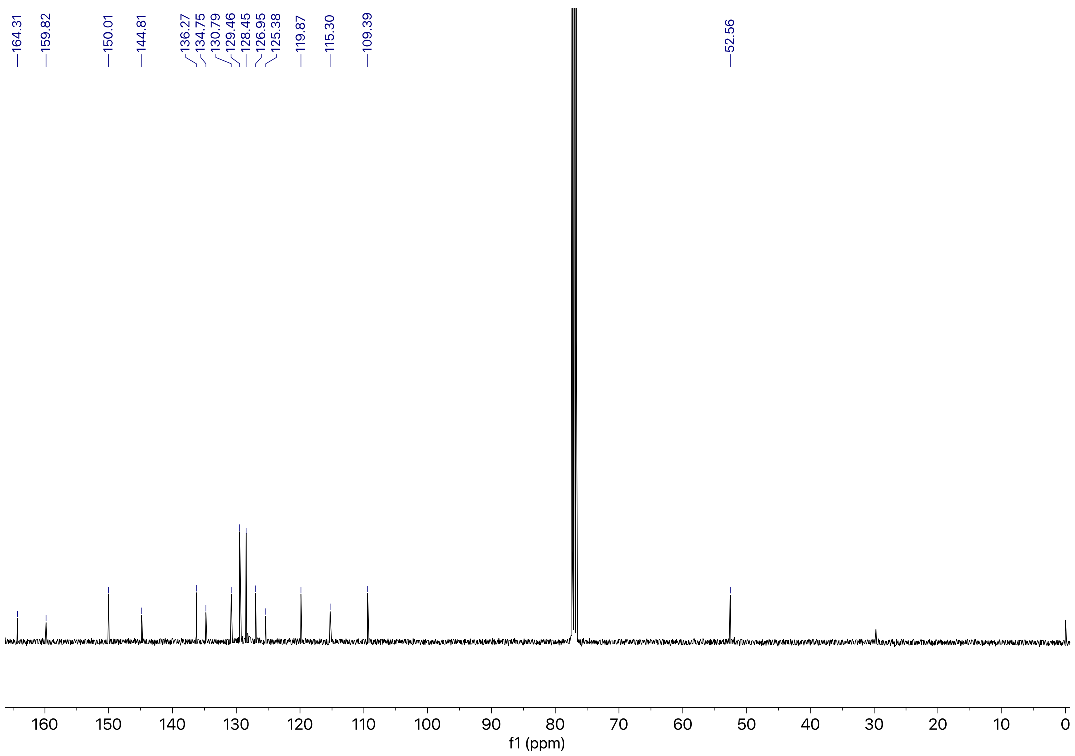

NMR data for methyl 8-(4-chlorophenyl)-7-hydroxyquinoline-6-carboxylate (**42**)

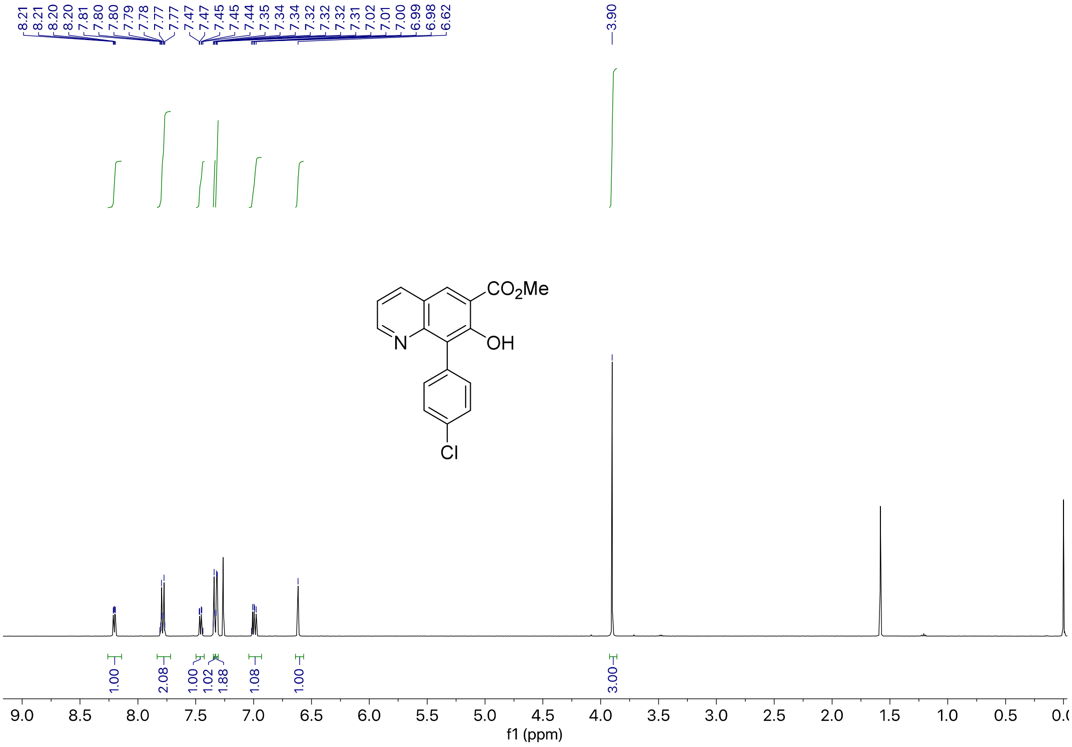

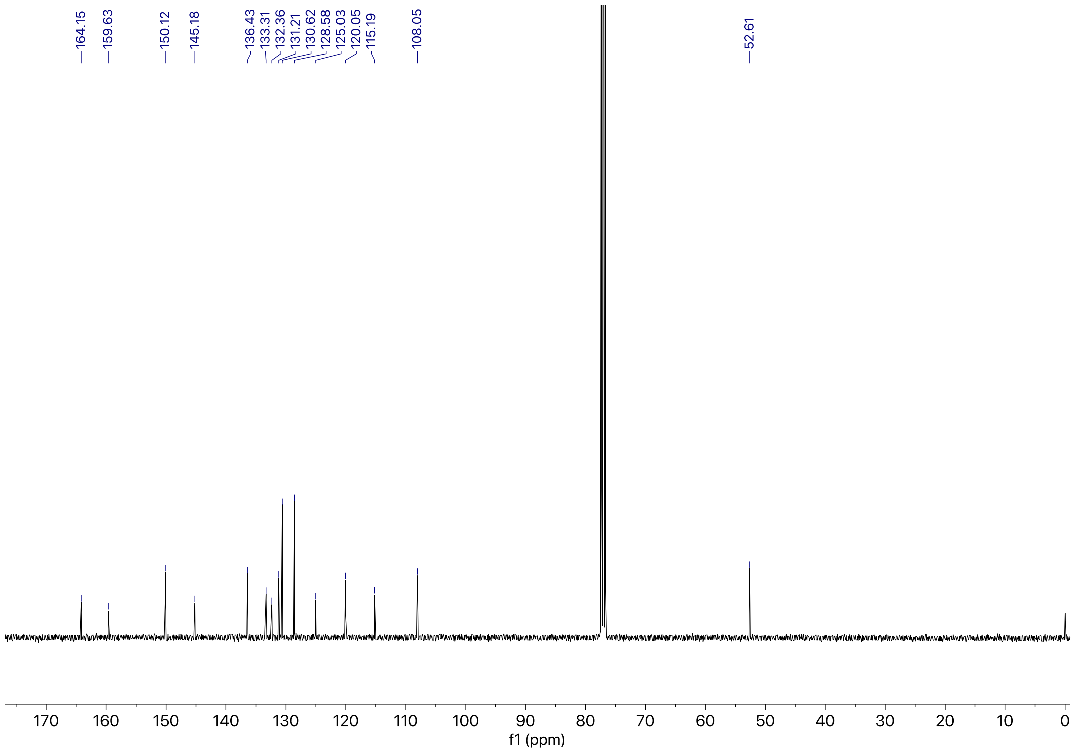

NMR data for methyl 7-hydroxy-8-(4-methylphenyl)quinoline-6-carboxylate (**43**)

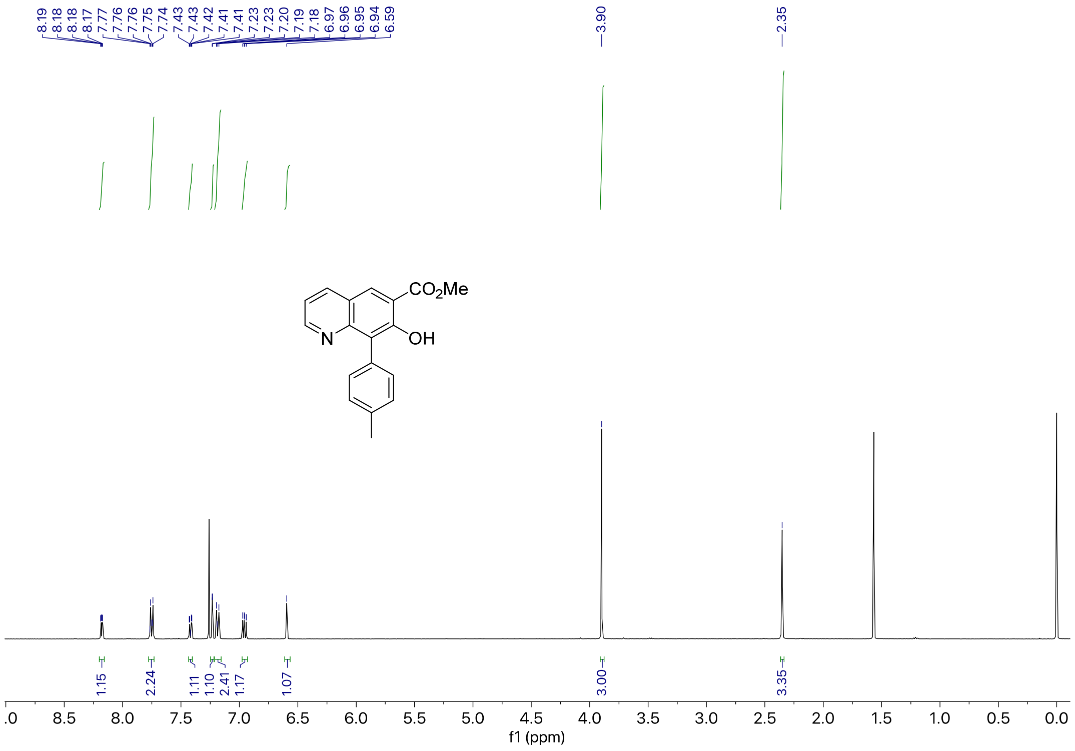

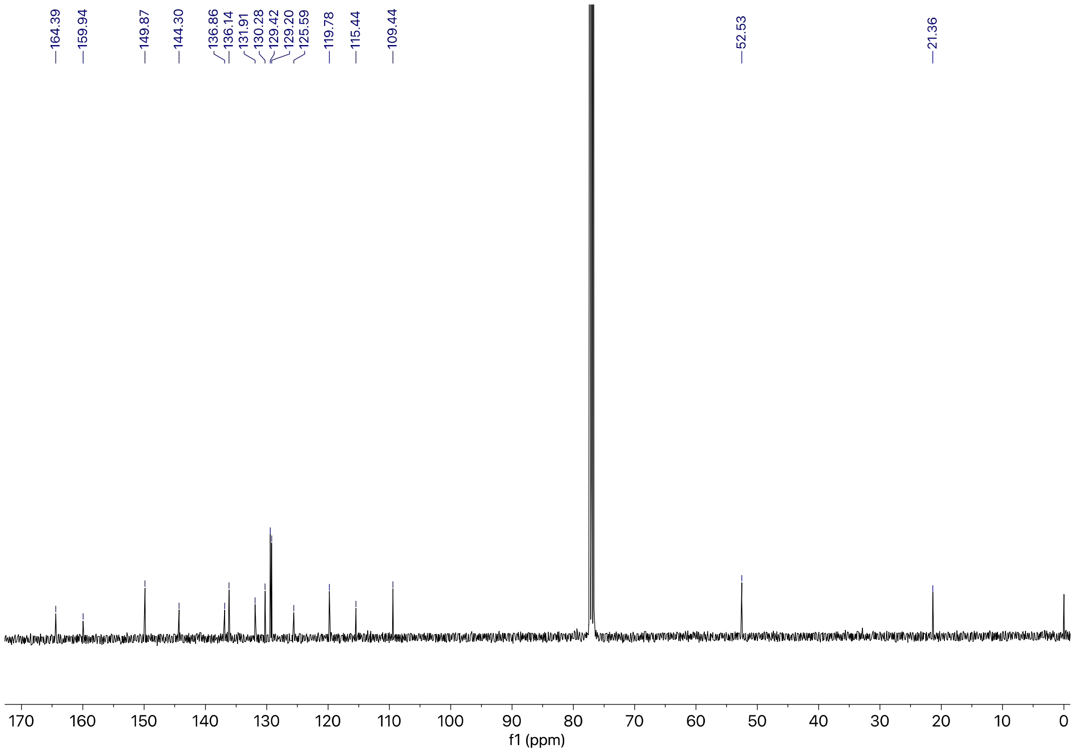

NMR data for methyl 7-hydroxy-8-(4-methoxyphenyl)quinoline-6-carboxylate (**44**)

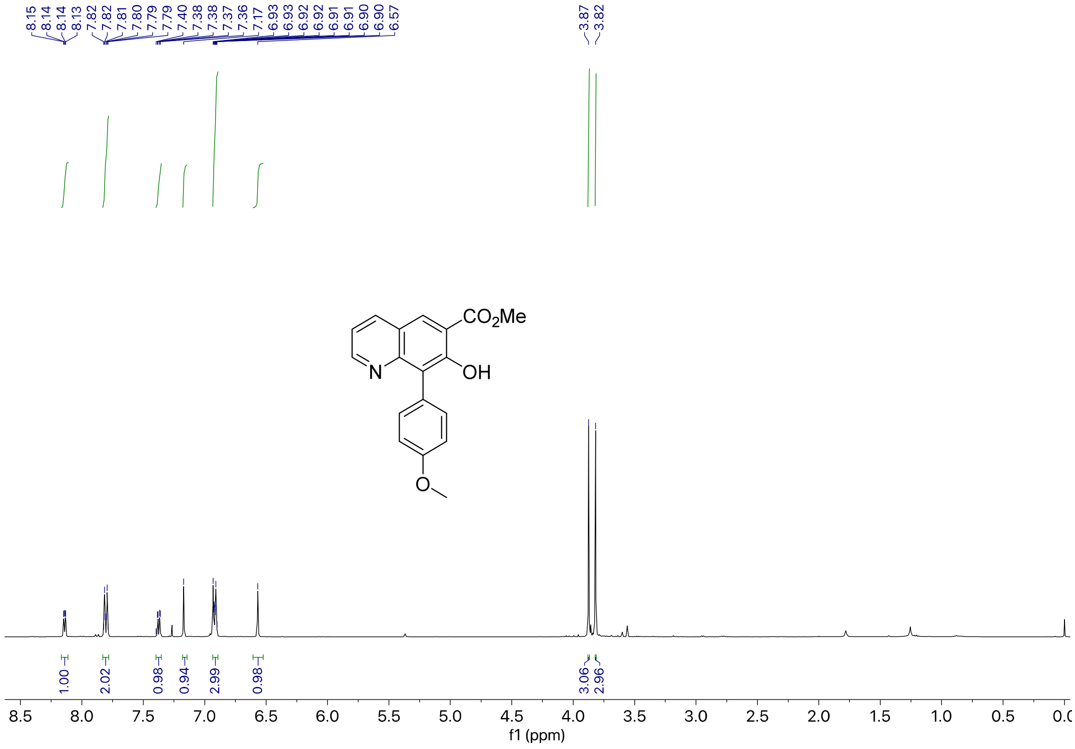

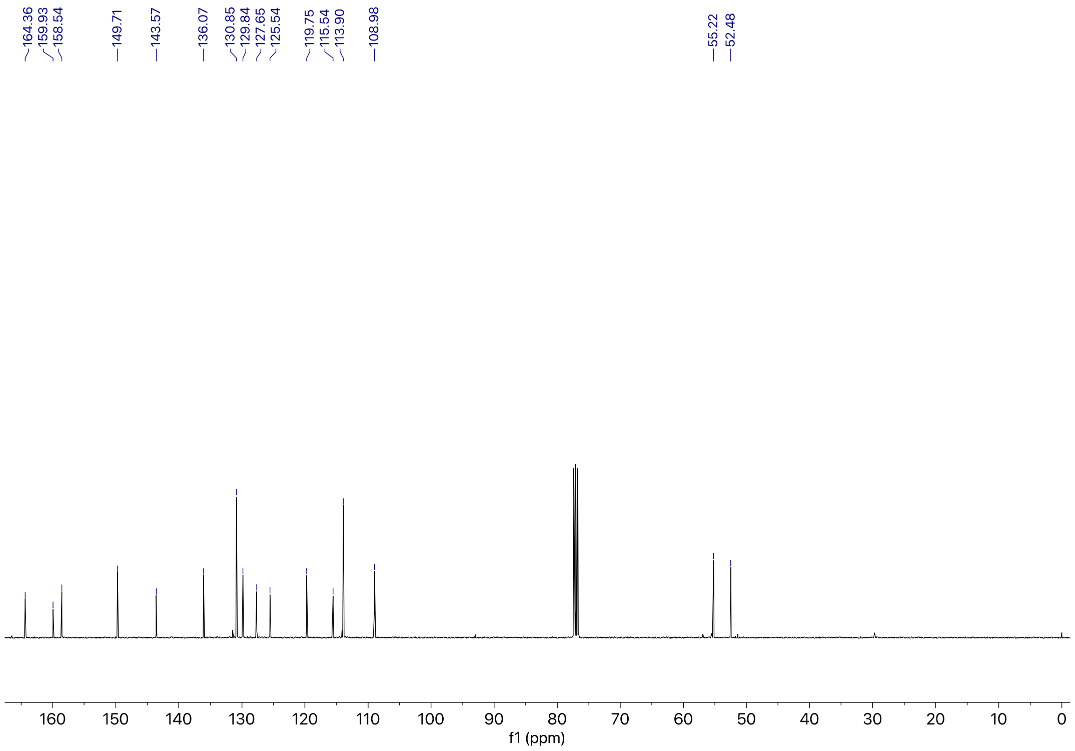
164.36
159.93
158.54
149.71
143.57
136.07
130.85
129.84
127.65
125.54
119.75
115.54
113.90
108.98
55.22
52.48
160
150
140
130
120
110
100
90
80
70
60
50
40
30
20
10
0
f1 (ppm)
